# Gasdermin E deficiency attenuates acute kidney injury by inhibiting pyroptosis and inflammation

**DOI:** 10.1038/s41419-021-03431-2

**Published:** 2021-02-01

**Authors:** Weiwei Xia, Yuanyuan Li, Mengying Wu, Qianqian Jin, Qian Wang, Shuzhen Li, Songming Huang, Aihua Zhang, Yue Zhang, Zhanjun Jia

**Affiliations:** 1 Nanjing Key Laboratory of Pediatrics, Children’s Hospital of Nanjing Medical University, 210008 Nanjing, China; 2 Department of Nephrology, Children’s Hospital of Nanjing Medical University, Guangzhou Road #72, 210008 Nanjing, China; 3 Jiangsu Key Laboratory of Pediatrics, Nanjing Medical University, 210029 Nanjing, China

**Keywords:** Acute kidney injury, Acute inflammation

## Abstract

Pyroptosis, one kind of inflammatory regulated cell death, is involved in various inflammatory diseases, including acute kidney injury (AKI). Besides Gasdermin D (GSDMD), GSDME is a newly identified mediator of pyroptosis via the cleavage of caspase-3 generating pyroptotic GSDME-N. Here, we investigated the role of GSDME in renal cellular pyroptosis and AKI pathogenesis employing GSDME-deficient mice and human tubular epithelial cells (TECs) with the interventions of pharmacological and genetic approaches. After cisplatin treatment, GSDME-mediated pyroptosis was induced as shown by the characteristic pyroptotic morphology in TECs, upregulated GSDME-N expression and enhanced release of IL-1β and LDH, and decreased cell viability. Strikingly, silencing GSDME in mice attenuated acute kidney injury and inflammation. The pyroptotic role of GSDME was also verified in human TECs in vitro. Further investigation showed that inhibition of caspase-3 blocked GSDME-N cleavage and attenuated cisplatin-induced pyroptosis and kidney dysfunction. Moreover, deletion of GSDME also protected against kidney injury induced by ischemia-reperfusion. Taken together, the findings from current study demonstrated that caspase-3/GSDME-triggered pyroptosis and inflammation contributes to AKI, providing new insights into the understanding and treatment of this disease.

## Introduction

Acute kidney injury (AKI) is a common clinical complication characterized by rapid decline in kidney function^1^. It is a global public health concern, with high morbidity and mortality, leading to ~1.7 million deaths per year^2^. Despite partial renal recovery being possible, AKI has always been linked to prolonged hospitalization and progression to end-stage renal disease owing to the lack of satisfactory therapeutic strategies^3,4^. Cis-diamminedichloroplatinum (cisplatin) is an effective agent for treating solid tumors. However, its clinical use is limited by its serious toxic side effects; nephrotoxicity is the most common side effect and is noticed in ~25–40% of patients undergoing cisplatin therapy^5^. In addition, ischemia-reperfusion (IR) is a common insult leading to AKI. During AKI, renal tubular cell death occurs. Necrosis and apoptosis are the two major forms of cell death contributing to decline in renal function. Accumulating evidence has shown that strategies targeting apoptosis help extenuate renal tubular injury caused by cisplatin^6,7^. Several types of necrosis have been recently identified as regulated processes and are mediated by diverse molecules^8^. Despite this, the factors triggering kidney injury need to be investigated in detail.

Pyroptosis, a type of inflammatory caspase-dependent necrotic cell death, occurs exclusively in macrophages and dendritic cells^9^. It also exists in some other cell types, including hepatocytes^10^ and tubular epithelial cells (TECs). In the kidney, pyroptosis is reportedly induced by cadmium^11^, contrast^12^, renal ischemia-reperfusion^13^, and unilateral ureteral obstruction^14^ and is characterized by the activation of inflammatory caspases and release of IL-1β. Pyroptosis is mainly activated by inflammatory caspases, leading to cell swelling, pore formation, cell membrane disruption, and consequent inflammatory cytokine release. Canonical inflammatory caspase-1; non-canonical caspase-11, −4, and −5; or other factors, such as ELANE or caspase-8, cleave gasdermin D (GSDMD) to generate N-terminal domain gasdermin D (GSDMD-N), which then oligomerizes and forms a membrane pore, allowing the release of proinflammatory cytokines to the extracellular space^15,16^. Due to the similar structure of gasdermin domain, the pore-forming function may be also anticipated for some other gasdermin superfamily members.

GSDME, also called DFNA5, was first found to be associated with autosomal dominant nonsyndromic deafness^17^. Meanwhile, GSDME expression silencing in multiple cancer cells via DNA methylation of the promoter region related it to tumor metastasis and chemotherapy resistance, indicating its role as a tumor suppressor. This role may be associated with phagocytosis of tumor-associated macrophages, tumor-infiltrating natural-killer cells, and CD8^+^ T lymphocytes^18,19^. In addition, GSDME can be cleaved by caspase-3, an acknowledged apoptotic caspase, in GSDME-expressing tumor cells and GSDME-negative cells with extrinsic overexpressed GSDME to induce pyroptosis^20^. Moreover, GSDME has been recently reported to be cleaved by killer cell granzyme B at the same site as caspase-3 in GSDME-expressing cells, but in a caspase-3-independent manner^18^. GSDME-knockout mice subjected to chemotherapy also showed reduced tissue damage compared with WT mice subjected to the same treatment, suggesting an extra role of GSDME in addition to tumor suppression^20^. Pyroptosis participates in various kidney diseases, as evidenced by the activation of inflammatory caspases, massive release of IL-1β, or increased cleavage of GSDMD^21,22^. However, the role of GSDME in AKI remains to be illustrated. Moreover, the detailed mechanisms are still unknown. Here, we used GSDME-deficient mice to determine the role of GSDME in experimental AKI mouse models.

## Results

### Cisplatin induced pyroptosis in mice and human TECs

**Fig. 1:**
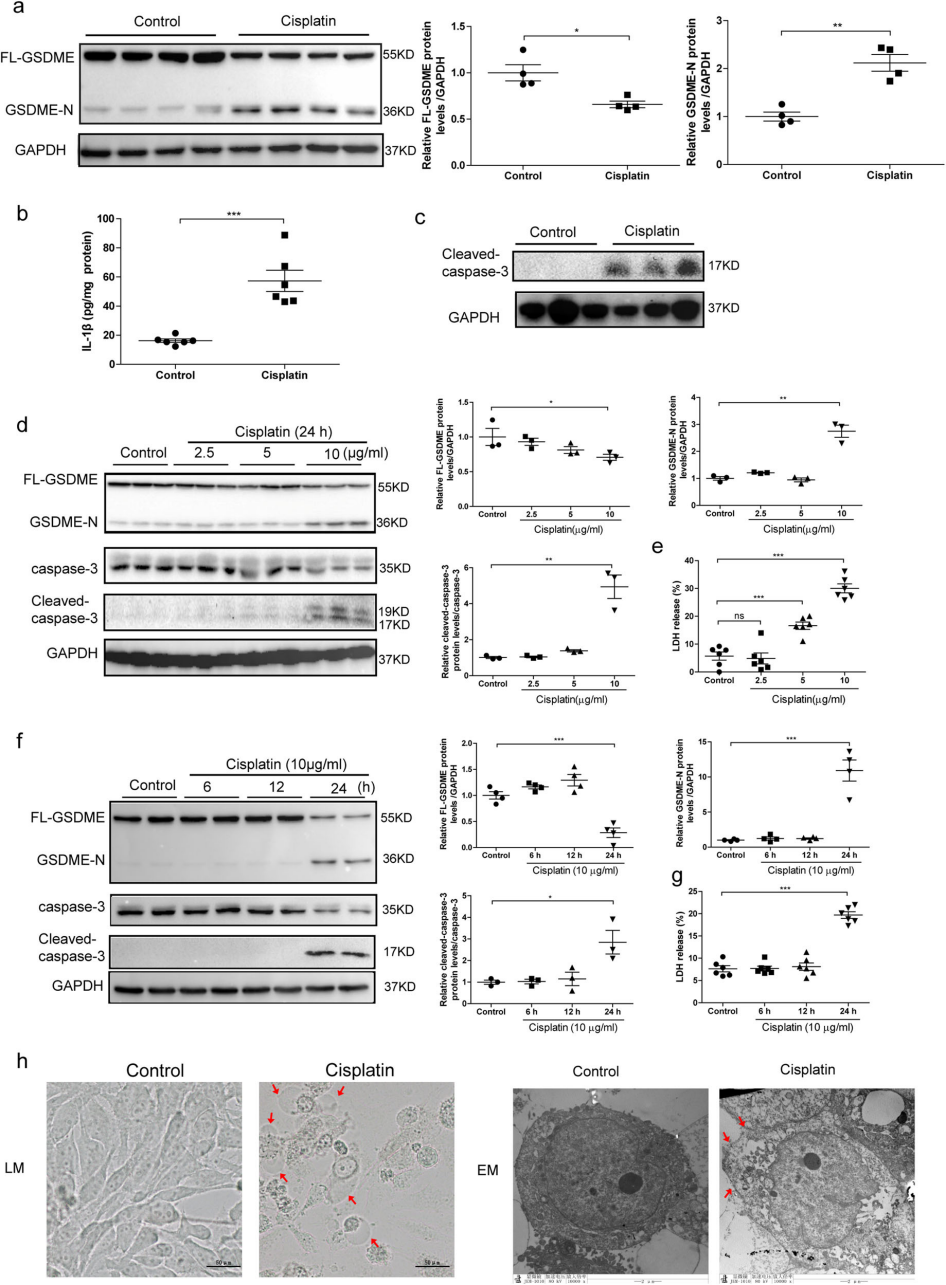
Cisplatin activated GSDME and induced pyroptosis in kidney tissues and human renal epithelial cells. Mouse kidney tissues and human renal epithelial cells subjected to cisplatin were used to determine the expression of GSDME and the existence of pyroptosis. **a** Protein level of FL-GSDME and GSDME-N in mouse kidneys subjected to cisplatin (*n* = 4). **b** Renal IL-1β production in mice subjected to cisplatin (*n* = 6). **c** Protein level of cleaved caspase-3 in mouse kidneys after cisplatin treatment (*n* = 3). **d** Protein levels of FL-GSDME, GSMDE-N, caspase-3, and cleaved caspase-3 in cells treated with different doses of cisplatin for 24 h (*n* = 3). **e** LDH release in cells treated with different doses of cisplatin for 24 h (*n* = 6). **f** Protein level of FL-GSDME, GSMDE-N, caspase-3, and cleaved caspase-3 in cells treated with 10 μg/mL of cisplatin for different time intervals (*n* = 3–4). **g** LDH release in cells treated with 10 μg/mL cisplatin for different time intervals (*n* = 6). **h** Representative bright-field (scale bar = 50 μm) and transmission electron microscopy (magnification × 10000; scale bar = 2 μm) of cells incubated with cisplatin or the corresponding control. Red arrowheads indicate large bubbles and pores emerging from the plasma membrane. *P < 0.05, **P < 0.01, and ***P < 0.001.

Pyroptosis has been illustrated to be involved in several kidney diseases^23,24^. Apart from GSDMD, an acknowledged executor of pyroptosis, GSDME was also recently identified to trigger pyroptosis in several diseases, especially in cancers^25^. To clarify whether GSDME involves in cisplatin-induced AKI, we measured the expression and cleavage of FL-GSDME in kidney tissue from cisplatin-treated mice. By immunohistochemistry, total GSDME protein was found to be expressed in renal tubules without obvious alteration after cisplatin treatment (data not shown). However, FL-GSDME was decreased by cisplatin, GSDME-N was significantly elevated (Fig. 1a). In addition, there was significant elevation in the renal production of IL-1β and cleaved caspase-3, which implements GSDME cleavage in kidneys with cisplatin treatment (Fig. 1b,c). Thus, GSDME-derived pyroptosis was possibly involved in cisplatin-induced AKI. To define the expression of GSDME in vitro, human TECs (HK-2) were treated with different doses of cisplatin for 24 h or with 10 μg/mL cisplatin for different time periods. In line with the results in vivo, GSDME-N level in HK-2 cells was significantly elevated with 10 μg/mL cisplatin for 24 h in parallel with the increased activation of caspase-3, although FL-GSDME expression decreased under this condition (Fig. 1d, f). LDH release is a marker of cell lysis and indicates the level of pyrotosis. Our results showed significant elevation of LDH release after treatment with 5 μg/mL cisplatin for 24 h, with higher promotion at 10 μg/mL (Fig. 1e, g). Morphologically, human TECs displayed cell swelling under light microscopy and massive pore formation of membranes under TEM in HK-2 cells after cisplatin treatment (Fig. 1h). Moreover, we also confirmed that the activity of caspase-1 and the cleavage of GSDMD were significantly promoted in HK-2 cells (Fig. S1).

### GSDME deletion attenuated cisplatin-induced kidney injury and inflammation in vivo

**Fig. 2:**
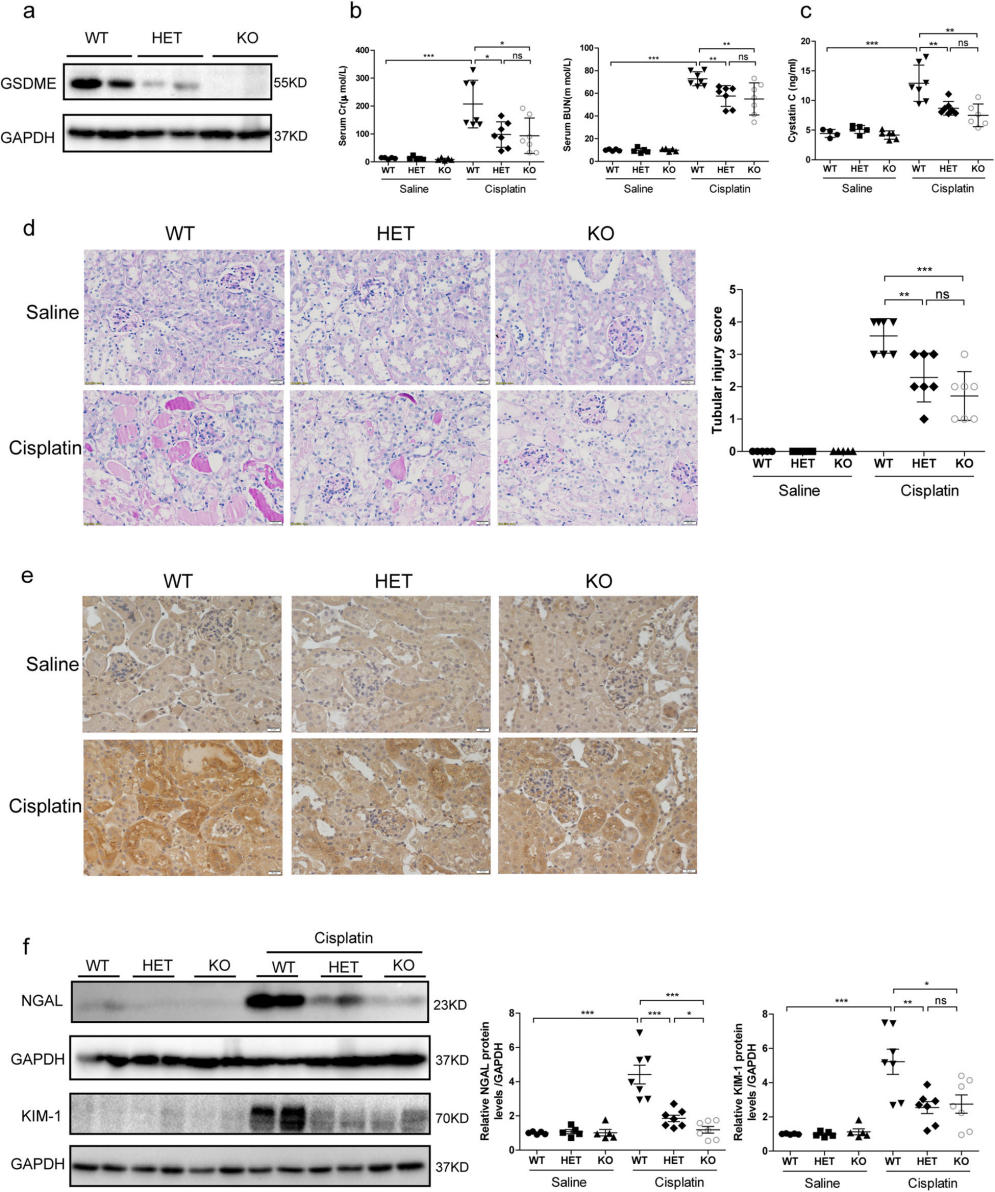
GSDME deletion attenuated cisplatin-induced renal function loss and pathological injury. WT, HET, and KO mice (12 weeks) were subjected to 25 mg/kg of cisplatin or the same volume of saline (Saline group, *n* = 5; Cisplatin group, *n* = 7). **a** Expression of GSDME in mouse kidneys by immunoblotting (*n* = 2). **b**, **c** Serum Cr, BUN, and Cystatin C levels of mice. **d** PAS staining and the associated renal tubular injury score analysis (scale bar = 20 μm). **e** Renal protein level of NGAL in kidney tissues, as detected by immunohistochemistry (scale bar = 20 μm). **f** Immunoblotting of NGAL and KIM-1 in kidney tissues. *P < 0.05, **P < 0.01, and ***P < 0.001.

**Fig. 3:**
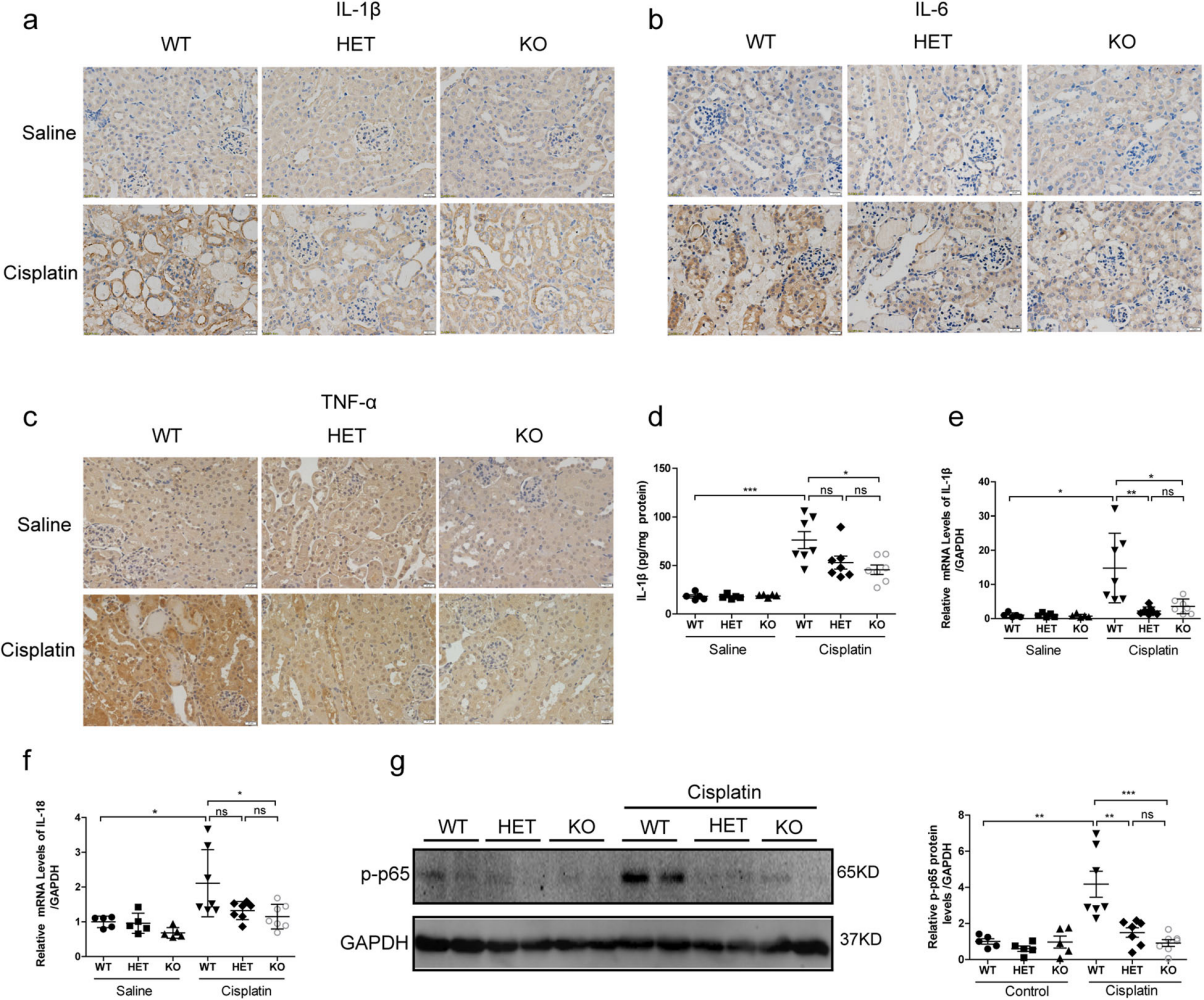
GSDME deletion extenuated cisplatin-induced inflammation in kidney. **a**–**c** Immunohistochemical staining of IL-1β, IL-6, and TNF-α (scale bar = 20 μm). **d** Renal production of IL-1β detected by ELISA. **e**, **f** Transcriptional levels of IL-1β and IL-18 in mouse kidney tissues. **g** p-p65 protein levels in mouse kidney tissues. *P < 0.05, **P < 0.01, and ***P < 0.001.

GSDME deficiency was reported to protect mice from chemotherapy-induced tissue damage and inflammation^26^. Pyroptotic evidence in kidney tissues subjected to cisplatin has been reported. However, the role of GSDME in cisplatin-induced AKI remains to be illustrated. Here, GSDME-deficient mice were used to investigate the role of GSDME in cisplatin-induced AKI. We first verified the deletion of GSDME in mouse kidneys (Fig. 2a). After intraperitoneal injection of cisplatin for 72 h, we observed significant recovery of renal function in mice with GSDME deletion, characterized by obvious reduction in serum Creatinine (Cr), Blood Urea Nitrogen (BUN), and Cystatin C levels (Fig. 2b, c). Interestingly, there was no significant difference in renal function between HET-Cisplatin and KO-Cisplatin mice with cisplatin treatment, indicating that moderate blockade of GSDME could also protect mice from cisplatin-induced kidney injury. In accordance, kidney pathology of mice in HET-Cisplatin group and KO-Cisplatin group showed less renal tubular injury than the WT-Cisplatin group, characterized by obvious attenuation of tubular dilatation, loss of brush border, tubular cell dropout, and cast formation (Fig. 2d). Meanwhile, renal protein levels of NGAL and KIM-1, which are markers of renal tubular injury, all declined in GSDME-deficient kidney tissues compared with those in the WT-Cisplatin kidney tissues (Fig. 2e, f). Additionally, TUNEL staining was performed to analyze the apoptosis of renal tubular cells. As shown in Fig. S2, the percentage of TUNEL-positive cells was strikingly increased in the kidneys of cisplatin-treated WT animals, which was reduced in the kidney tissues of GSDME-deficient mice. We also evaluated the role of GSDME in inflammation in cisplatin-induced AKI. As expected, renal IL-1β, IL-6, and TNF-α levels were significantly reduced in GSDME-deficient mouse kidneys, as measured by immunohistochemical staining and/or ELISA (Fig. 3a–d). Meanwhile, enhanced renal mRNA levels of IL-1β and IL-18 were also blocked in kidneys with GSDME deletion (Fig. 3e, f). Furthermore, a mitigation of p-p65 protein expression was observed in HET-Cisplatin and KO-Cisplatin kidneys compared with the WT kidneys subjected to cisplatin (Fig. 3g). All these data indicate that GSDME-associated pyroptosis could further aggravate the inflammatory response possibly via a positive feedback loop.

### GSDME-N promoted cisplatin-induced cell injury and pyroptosis in vivo and in vitro

**Fig. 4:**
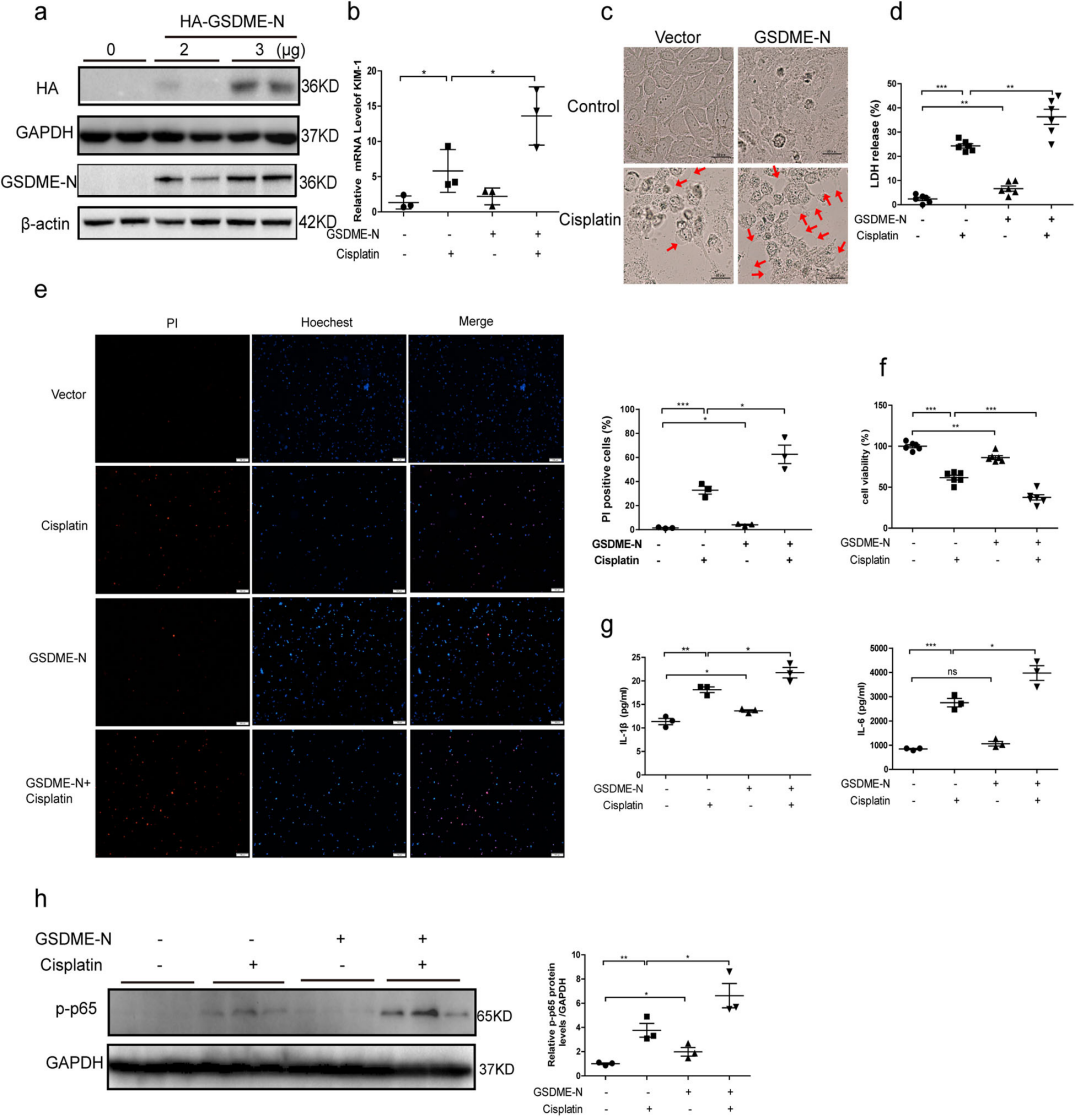
GSDME-N promoted cisplatin-induced cell injury and pyroptosis in human TECs. Human TECs (HK-2) were transfected with GSDME-N (residues 1–270aa) overexpressing plasmid and subsequently treated with cisplatin (10 μg/mL) for 24 h. **a** Protein levels of HA-GSDME-N in cells by immunoblotting with HA and GSDME antibody. **b** Transcriptional levels of KIM-1 (*n* = 3). **c** Representative bright-field microscopic images of cells treated with cisplatin in the presence or absence of GSDME-N. Red arrowheads indicate large bubbles from the plasma membrane (scale bar = 50 μm). **d** LDH release of cells transfected with GSDME-N in the presence of cisplatin treatment (*n* = 6). **e** PI staining of cells with GSDME-N overexpression (scale bar = 100 μm; *n* = 3). **f** CCK-8 assay was performed to assess the effect of GSDME-N on cell viability (*n* = 6). **g** ELISA results of IL-1β and IL-6 in cultured medium of cells with GSDME-N overexpression (*n* = 3). **h** The effect of GSDME-N on p-p65 protein levels (*n* = 3). *P < 0.05, **P < 0.01, and ***P < 0.001.

The previous research has identified that caspase-3 cleaves GSDME at 267DMPD270 in humans. GSDME-N with pore-forming activity perforated cell membranes to induce pyroptosis in several cell lines^20^. To further confirm the effect of GSDME-N-mediated pyroptosis in cisplatin-induced AKI, we used the tail vein rapid injection method to express the GSDME-N plasmids (residues 1–270aa) in kidney. Briefly, 80 μg of control vectors and GSDME-N plasmids were injected into WT and GSDME-deficient mice within 10 s via tail vein. After 36 h, mice were administrated with cisplatin for 72 h. We found that the protection on renal function and tubular injury in GSDME-deficient mice was abolished by GSDME-N overexpression (Fig. S3a–f). We also transfected human TECs with GSDME-N overexpressing plasmids (residues 1–270aa) to further identify the potential effect of GSDME-N in vitro. In cells with GSDME-N overexpressing, KIM-1 was significantly upregulated (Fig. 4a, b). The dying cells with overexpressed GSDME-N appeared to be evidently swelling, with characteristic large bubbles from the plasma membrane compared with cells transfected with the corresponding control (Fig. 4c). Overexpression of GSDME-N further promoted increased LDH release and nuclear PI staining induced by cisplatin in human TECs (Fig. 4d, e). In addition, cell viability of human TECs was significantly decreased after GSDME-N overexpression (Fig. 4f). Furthermore, IL-1β and IL-6 release were both increased in cells transfected with GSDME-N-overexpressing plasmid (Fig. 4g). Additionally, phosphorylated p65 was elevated in GSDME-N-overexpressing cells (Fig. 4h). All these results suggest that GSDME-N aggravated cisplatin-induced human renal tubular cell injury by inducing pyroptosis and further amplifying inflammatory response, which could be secondary to the GSDME-mediated pyroptosis.

### GSDME is involved in cisplatin-induced cell injury and pyroptosis in human TECs

**Fig. 5:**
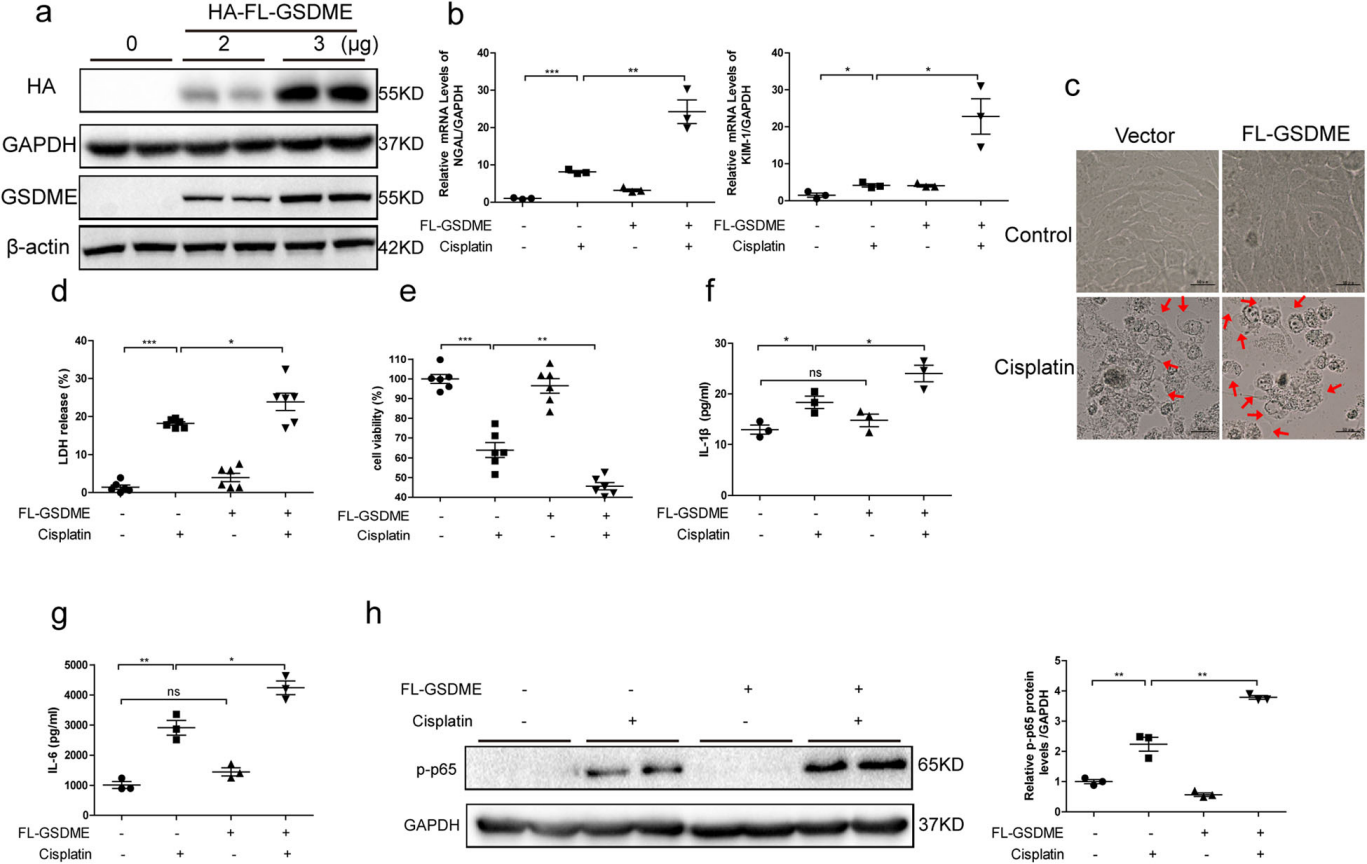
FL-GSDME aggravated cisplatin-induced cell injury and pyroptosis in human TECs. Human TECs (HK-2) were transfected with FL-GSDME or the corresponding control and subsequently treated with cisplatin (10 μg/mL) for 24 h. **a** Protein expression of HA-FL-GSDME with HA and GSDME antibody. **b** Transcriptional levels of NGAL and KIM-1 (*n* = 3). **c** Representative bright-field microscopic images of cells. Red arrowheads indicate large bubbles from the plasma membrane (scale bar = 50 μm). **d** LDH release in cells overexpressing FL-GSDME (*n* = 6). **e** Effect of FL-GSDME on the cell viability (*n* = 6). **f**, **g** IL-1β and IL-6 released in culture medium of cells. **h** Effect of FL-GSDME on p-p65 protein levels (*n* = 3). *P < 0.05, **P < 0.01, and ***P < 0.001.

**Fig. 6:**
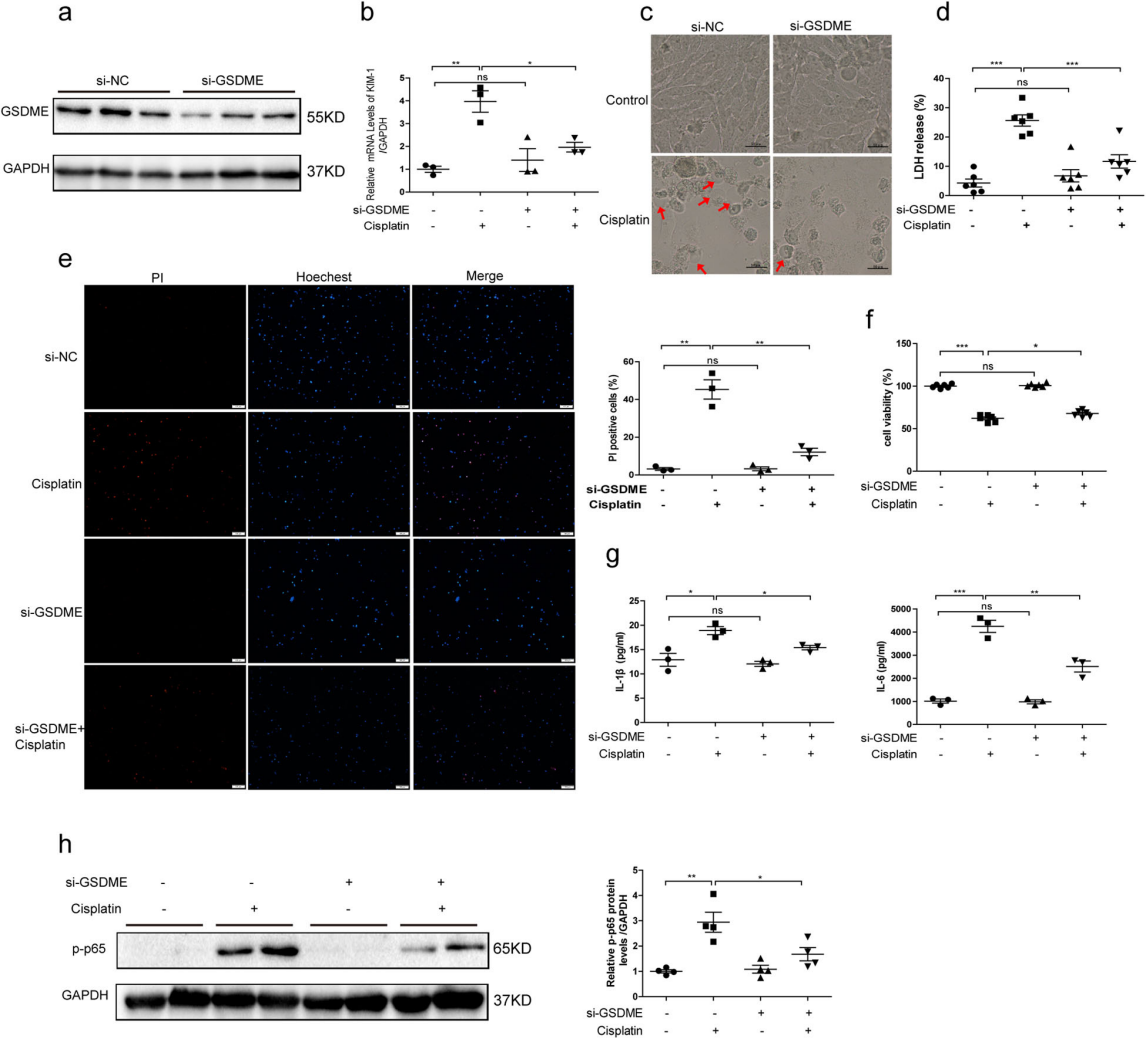
GSDME knockdown relieved cisplatin-induced cell injury and pyroptosis in human TECs. Human TECs (HK-2) were transfected with FL-GSDME siRNA or the negative control and subsequently treated with cisplatin (10 μg/mL) for 24 h. **a** Protein level of FL-GSDME in human TECs after transfection with FL-GSDME siRNA (*n* = 3). **b** Transcriptional levels of KIM-1 (*n* = 3). **c** Representative bright-field microscopic images of cells. Red arrowheads indicate large bubbles from the plasma membrane (scale bar = 50 μm). **d** Role of GSDME silencing on LDH release (*n* = 6). **e** PI staining of cells with GSDME knockdown (scale bar = 100 μm; *n* = 3). **f** Role of GSDME siRNA on cell viability (*n* = 6). **g** IL-1β and IL-6 release in cells (*n* = 3). **h** Effect of GSDME siRNA on p-p65 protein levels (*n* = 4). *P < 0.05, **P < 0.01, and ***P < 0.001.

Regarding the relief of renal tubular injury in GSDME-deficient mice, we further investigated the functions of FL-GSDME in cisplatin-treated human TECs using overexpressing plasmid and siRNAs of FL-GSDME. Former studies have shown that GSDME-N and not GSDME-C or FL-GSDME bond liposomes and perforate membranes. Consistent with the reported results, FL-GSDME had little effect on pyroptosis in human TECs without the treatment of cisplatin, represented via minimal changes in cell morphology. However, in the presence of cisplatin, FL-GSDME provided similar functions as GSDME-N in inducing pyroptosis. Overexpression of FL-GSDME further enhanced the expressions of NGAL and KIM-1 induced by cisplatin (Fig. 5a, b). Meanwhile, more pyroptotic cells with increased LDH release and decreased cell viability were observed in the FL-GSDME+ Cisplatin group, possibly mediated by the additional production of GSDME-N cleaved by caspase-3 (Fig. 5c–e). FL-GSDME also played an important role in cytokine release, such as IL-1β and IL-6 (Fig. 5f, g), along with enhanced expression of phosphorylated p65 (Fig. 5h). After knocking down GSDME in human TECs, in accordance with the results in GSDME-deficient mice, the transcriptional level of KIM-1 was decreased (Fig. 6a, b). Meanwhile, ablation of GSDME partially rescued the pyroptotic morphology of cells induced by cisplatin, characterized by the decreased ration of swelling cells (Fig. 6c). Due to lesser lysis of cells, the increased LDH release and PI staining ratio were also blocked in cells with GSDME silencing, consistent with the increased cell viability (Fig. 6d–f). In addition, GSDME silencing inhibited inflammatory responses, as shown by decreased IL-1β and IL-6 release and phosphorylated p65 expression (Fig. 6g, h). The above results further confirmed that GSDME could be involved in the pathogenesis of cisplatin-induced renal tubular injury and inflammation.

### Caspase-3 cleaved GSDME in cisplatin-treated human TECs and contributed to the AKI

**Fig. 7:**
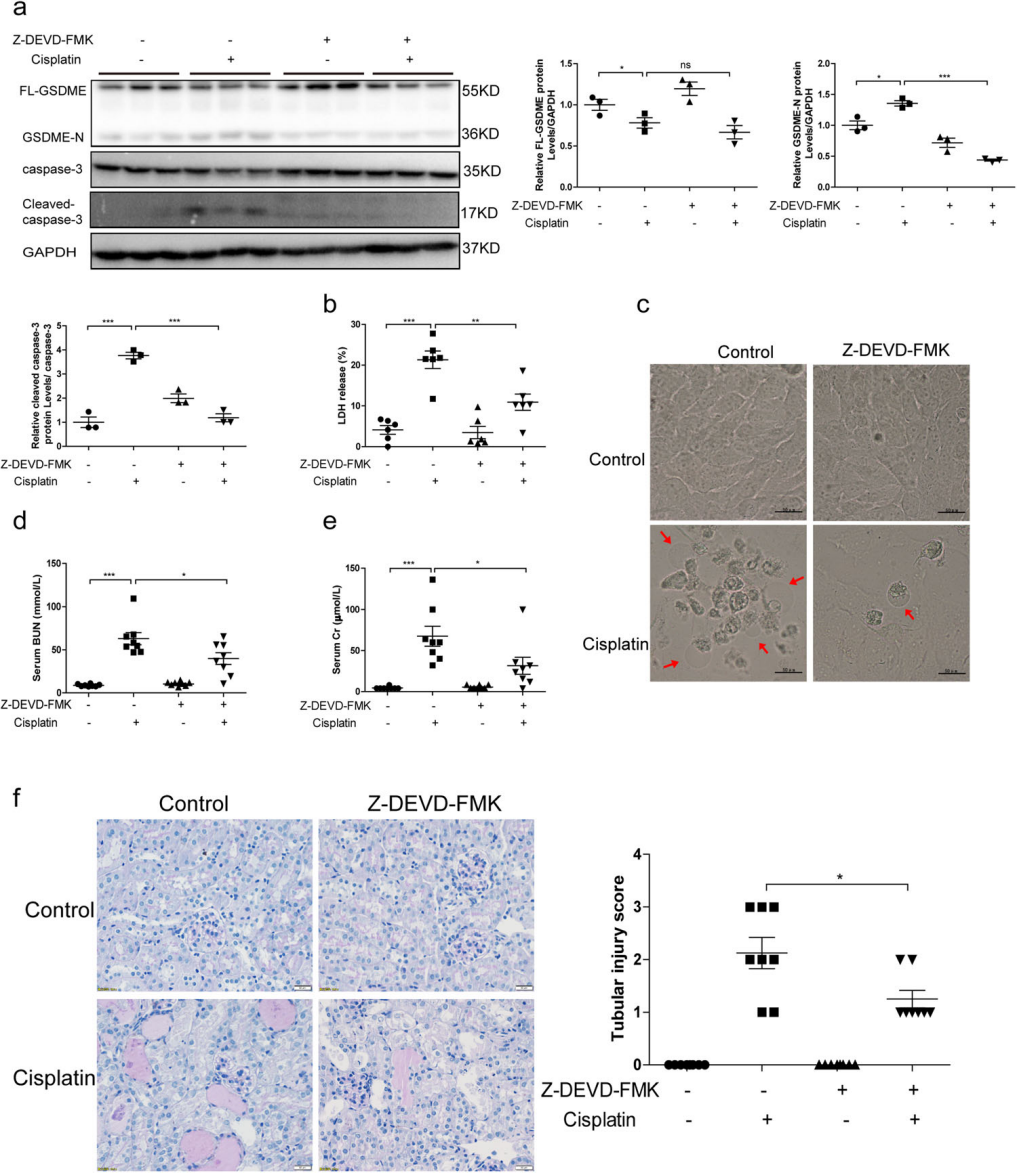
Caspase-3-specific inhibitor Z-DEVD-FMK relieved cisplatin-induced renal tubular cell injury and pyroptosis. Human TECs (HK-2) and mouse kidney tissues subjected to Z-DEVD-FMK were used to determine the role of caspase-3 in GSDME cleavage and AKI progression. **a** Protein levels of FL-GSDME, GSDME-N, caspase-3, and cleaved caspase-3 detected by immunoblotting (*n* = 3). **b** Effect of Z-DEVD-FMK on LDH release (*n* = 6). **c** Representative bright-field microscopic images (scale bar = 50 μm) of cells. Red arrowheads indicate large bubbles from the plasma membrane. **d** Serum BUN of AKI mice subjected to Z-DEVD-FMK (*n* = 8). **e** Serum Cr of AKI mice subjected to Z-DEVD-FMK (*n* = 8). **f** PAS staining (scale bar = 50 μm) and associated renal tubular injury score of mice administered with daily injection of Z-DEVD-FMK (*n* = 8). *P < 0.05, **P < 0.01, and ***P < 0.001.

GSDME was reportedly cleaved by caspase-3 in several cell lines, such as gastric cancer cells and cardiomyocytes^27^. To investigate whether GSDME was cleaved in a caspase-dependent manner in TECs, the pan caspase inhibitor Z-VAD-FMK was applied to cisplatin-treated cells. Treatment of Z-VAD-FMK significantly downregulated the levels of GSDME-N and cleaved caspase-3 (Fig. S4a–d). Meanwhile, LDH release was decreased by Z-VAD-FMK in line with the improved cell viability of human TECs (Fig. S4e, f). In accordance, the inhibition of caspases repressed the ratio of swelling cells that was induced by cisplatin (Fig. S4g). Furthermore, siRNAs of caspase-3 and Z-DEVD-FMK, a specific inhibitor of caspase-3, were both employed to identify the role of caspase-3 in this experimental setting. We observed both genetic silencing and pharmacological inhibition of caspase-3 reduced GSDME-N protein level (Fig. S5a–e and Fig. 7a). Simultaneously, enhanced LDH release and restrained cell viability caused by cisplatin were improved in cells with caspase-3 inhibition (Fig. S5f, g and Fig. 7b), along with ameliorated cell swelling (Fig. S5h and Fig. 7c). In addition, we observed significant relief of renal injury in cisplatin-induced AKI mice that were intraperitoneally injected with Z-DEVD-FMK (Fig. 7d–f). Together, our results suggest that caspase-3 could cleave GSDME to induce pyroptosis and AKI to some extent besides proapoptotic action.

### GSDME deletion ameliorated renal ischemia-reperfusion injury

**Fig. 8:**
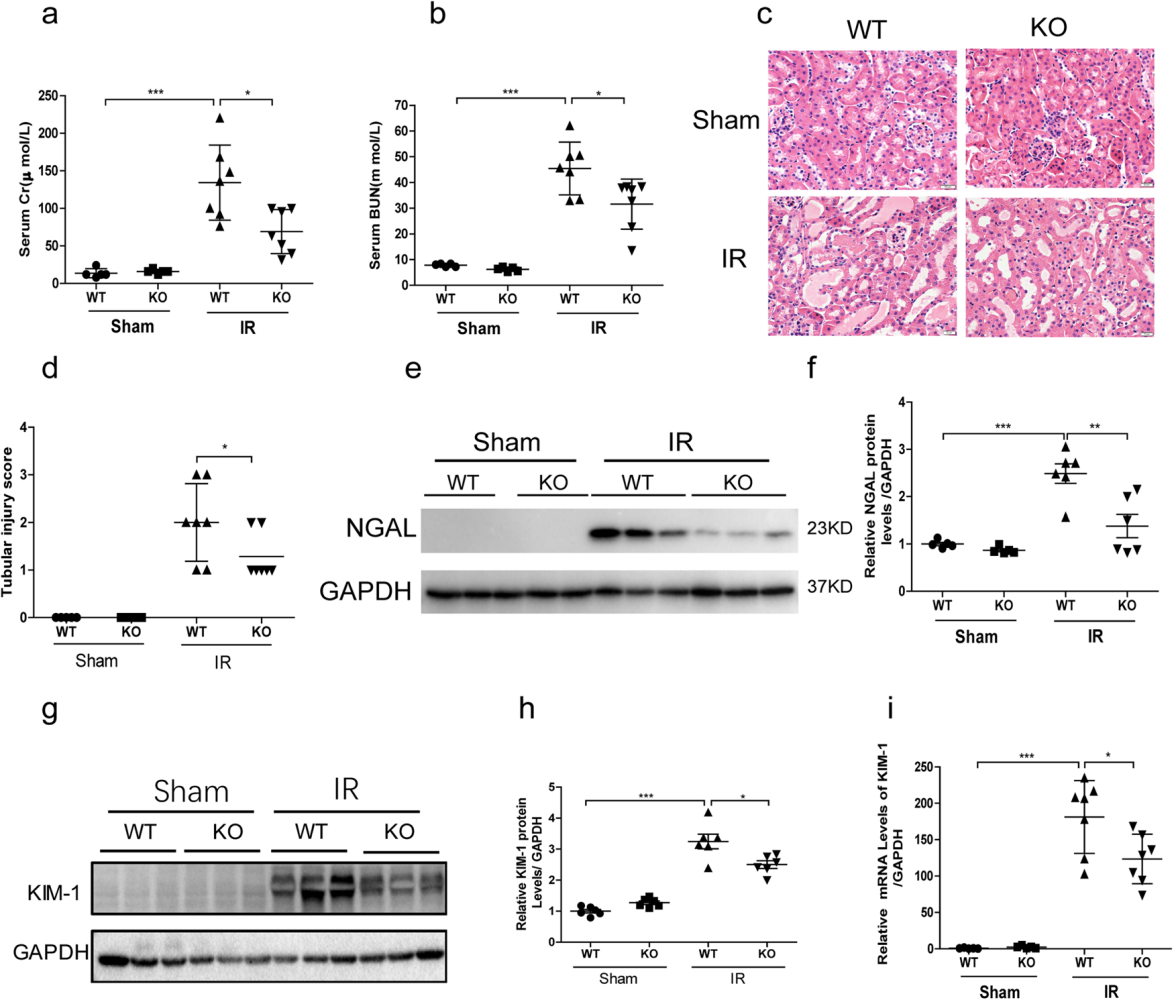
Ischemia-reperfusion-induced acute kidney injury was attenuated in GSDME deletion mice. GSDME KO and WT mice (8–12 weeks) underwent renal ischemia-reperfusion or the same procedure, without renal vessel occlusion (Sham group, *n* = 5; IR group, *n* = 7). **a**, **b** Serum Cr and BUN levels of mice. **c** HE staining of kidney sections (scale bar = 20 μm). **d** Renal tubular injury score of mice. **e** Renal protein level of NGAL. **f** Quantitative analysis of NGAL protein level from western blotting. **g** Renal protein level of KIM-1. **h** Quantitative analysis of KIM-1 protein level from western blotting. **i** Transcriptional levels of KIM-1 in mouse kidney. *P < 0.05, **P < 0.01, and ***P < 0.001.

Regarding the pathogenic role of GSDME in cisplatin-induced AKI, we further evaluated the role of GSDME in another AKI model. After IR, there were increases of serum Cr and BUN in WT-IR mice, which was significantly lowered in GSDME KO mice (Fig. 8a, b). Mice subjected to renal IR displayed severe kidney injury, characterized by obstructing granular cast formation and epithelial cell sloughing, whereas GSDME deficiency improved these pathological damages (Fig. 8c, d). Meanwhile, GSDME deficiency decreased the expression levels of NGAL and KIM-1 (Fig. 8e–i). These results offered additional evidence that GSDME might be a common therapeutic target for treating different types of AKI.

## Discussion

AKI is associated with renal tubular cell death and inflammation, both of which eventually lead to decline in renal function. Over the past decades, apoptosis and necrosis have been identified to contribute to AKI progression. Recently, regulated cell death, such as necroptosis, ferroptosis, and pyroptosis, has gained considerable attention. Among these, pyroptosis is considered the most common inflammatory cell death pathway^28^.

Previous studies have demonstrated that the activated inflammatory caspases mediated pyroptosis in response to various stimuli, characterized by the pore formation of cytomembranes and the release of proinflammatory cellular contents, such as IL-1β^29,30^. The GSDMD-N fragment is an executor of pyroptosis. In addition, GSDME, another member of gasdermin family, showed pore-forming capability, resulting in the amplification of inflammatory responses and leading to tissue damage^31^. Several studies have shown the definite role of GSDME in tumor growth and chemotherapy resistance^32,33^. Beyond cancer, GSDME deficiency also protects against chemotherapy-induced damage of the small intestine, lung, spleen, and other tissues^20^. Additionally, in adriamycin-treated cardiomyocytes and macrophages, GSDME and its cleaved form were induced, the knocking down of which alleviated adriamycin-induced pyroptosis^27^. However, little is known regarding the role of GSDME in AKI. In the present study, we aimed to investigate whether GSDME and GSDME-mediated pyroptosis were also involved in the pathogenesis of AKI. In cisplatin-induced AKI kidney tissues, elevated levels of GSDME-N were detectable, in parallel with increased renal IL-1β production. Renal tubular epithelial cells are the major organelle type responding to diverse injuries or irritations during AKI, and they participate in multiple proinflammatory cytokine release pathways. We tried to determine the functional participation of GSDME in cisplatin-induced renal tubular injury. In human TECs treated with cisplatin, pyroptotic cell morphology and increased LDH release were observed, accompanied by the upregulated level of GSDME-N. Due to the parallel level of GSDME-N and some other pyroptotic characteristics, GSDME-mediated pyroptosis might be a pathogenic mechanism influencing cisplatin-induced kidney injury.

We further employed GSDME-deficient mice to establish the experimental AKI model to verify the role of GSDME in cisplatin-induced kidney disease. GSDME-deficient mice treated with cisplatin showed relieved renal dysfunction and low kidney injury indexes, reflected by improved histopathologic indices and downregulated expressions of kidney injury markers compared to those in the WT mice with cisplatin treatment. This corroborates the effect of GSDMD in cisplatin-induced AKI^21^. Consistently, there was also a significant improvement of renal function in HET-Cisplatin mice. In addition to the cisplatin-induced AKI model, we generated the IR AKI model in GSDME-deficient mice and found that deletion of GSDME significantly protected renal tubules against ischemic injury. These results extended the potential of targeting GSDME in treating different types of AKI. A recently conducted study also provided evidence that GSDME promoted diabetic kidney disease by triggering pyroptosis, which is consistent with our results^34^. The role of FL-GSDME and its cleaved form was also determined in human TECs. By overexpressing the GSDME-N domain in vitro and in vivo, we detected significantly increased LDH release, positive PI staining, and enhanced swelling cell ratio accompanied by the upregulated expression of KIM-1 in vitro. More importantly, GSDME-N overexpression in kidney abolished the relief of renal function in GSDME deletion mice, suggesting that the pathogenic role of GSDME in cisplatin-induced AKI was mediated by increased GSDME-N expression, which further targeted the cell membrane and led to cell lysis. In accordance, we also observed extenuated cell injury and decreased pyroptotic cell death in GSDME knocking down cells, featured by reduced LDH release and pyroptotic morphology along with the improved cell viability and reduced NGAL and KIM-1 expressions.

GSDME-mediated pyroptosis was illustrated to play a cell-type-dependent role in inflammatory and autoimmune diseases, regulating the active IL-1β release by live cells and its passive shedding from dead cells once the cell explodes^35^. Meanwhile, the enhanced expression and release of IL-1β was increased in ischemic or drug-induced AKI, leading to further inflammatory cell recruitment and activation^36^. In our study, we also detected a functional role of GSDME in inflammation regulation, manifested on the significant reduced production of IL-1β, IL-6, and some other cytokines as well as the blockade of p-p65 expression in kidneys of GSDME-deficient mice and cells. Similar results were observed in human TECs transfected with GSDME-N, as shown by the accelerated release of IL-1β and IL-6 along with the activation of NF-κB pathway. Under AKI condition, caspase-1 could be activated to cleave pro-IL-1β, leading to the production of mature IL-1β^21,37^. Thus, increased GSDME-N could enhance pyroptosis, leading to the release of IL-1β, which could subsequently activate NF-κB pathway and increase the transcription of proinflammatory cytokines.

GSDME-N and not FL-GSDME was able to act on the plasma membrane in lipid composition, triggering extensive pyroptosis in 293 T cells^20^. In our study, we found that FL-GSDME overexpression significantly aggravated cell injury induced by cisplatin, characterized by enhanced expressions of NGAL and KIM-1. In the presence of cisplatin, cells with FL-GSDME overexpression showed a pyroptotic cell phenotype. Meanwhile, the release of cytokines was facilitated by upregulated FL-GSDME, which might be associated with the extra production of GSDME-N sourced from the cleavage of the exogenous FL-GSDME. Supporting our results, it was recently reported that GSDME cleavage generated a self-amplifying, positive feed-forward loop that GSDME-N fragment led to processing of FL-GSDME to generate additional GSDME-N fragments by permeabilizing mitochondria to augment caspase-3 activation^38^.

The role of caspase-3, a critical apoptotic executor of the caspase cascade, has been widely investigated. Caspase-3-dependent pyroptosis was well described in several cancer cells and inflammatory cells^20,39^. We detected an apparent increase in cleaved caspase-3 levels in both mouse kidney tissues and human TECs subjected to cisplatin, consistent with the increased GSDME-N, and provided a basis for the increased pyroptotic cellular features. With the use of Z-VAD-FMK, a pan caspase inhibitor, we detected significantly reduced level of GSDME-N, implying that cleavage of GSDME was dependent on the activation of caspases. By genetic knockout and specific pharmacological inhibition of caspase-3, we found that caspase-3-dependent cleavage of GSDME was essential for pyroptosis activation in human TECs. Blockade of cleaved caspase-3 led to less rupture of renal TECs and reduced release of inflammatory cytokines, which resulted in relieved renal function loss in vivo.

In summary, we demonstrated the critical role of GSDME-mediated pyroptosis in AKI. Caspase-3-dependent cleavage of GSDME is involved in membrane pore formation and cell lysis, further aggravating renal tubular injury and inflammation. Our study offers novel perspectives for understanding the pathogenesis and therapeutic targets of AKI. Moreover, findings from our and other groups showed that the deletion of GSDMD and GSDME were both effective in preventing pyroptosis in AKI, suggesting the existence of antipyroptotic mechanisms in kidney cells during pathology. Partial blockade of propyroptotic contributors could reset the balance of pro and antipyroptotic mechanisms, leading to the improvement of pyroptosis and injury.

## Methods

### Animal studies

GSDME-deficient (GSDME^−/−^, KO) C57BL/6 J mice were gifted by Professor Feng Shao of the National Institute of Biological Sciences. To construct the cisplatin-induced AKI animal model, the GSDME^−/−^(KO), GSDME^−/+^ (HET) mice, and their WT littermates (12-week-old, male mice) were randomly divided into six groups: WT+ Saline: *n* = 5, HET+ Saline: *n* = 5, KO+ Saline: *n* = 5, WT+ Cisplatin: *n* = 7, HET+ Cisplatin: *n* = 7, and KO+ Cisplatin: *n* = 7. A single dose of cisplatin (25 mg/kg, Sigma–Aldrich, MO, USA) or the same volume of saline was intraperitoneally injected into the WT, HET, and KO mice. Experimental mice were sacrificed after 72 h of cisplatin injection.

To validate the effect of GSDME-N on cisplatin-induced AKI, WT and GSDME^−/−^ (KO) mice (10–12 weeks, male) were randomly divided into four groups: WT+ Vector group: *n* = 8, WT+ Vector + Cisplatin group: *n* = 8, KO+ Vector + Cisplatin group: *n* = 10, KO+ GSDME-N + Cisplatin group: *n* = 10. Eighty microgram of control vector (pEGFP-N1) and GSDME-N (pEGFP-N1-GSDME-N) plasmids were injected into mice within 10 s via tail vein rapid injection method as described previously^37^. After 36 h, cisplatin was administrated for 72 h. All these mice were euthanized, and blood and renal tissues were collected for further analyses.

We also constructed the renal IR injury model as described previously^40^. Briefly, the experimental mice were anesthetized with 4.0% isoflurane and were placed on a heated surgical table to maintain the body temperature at 37 °C. After exposing the kidneys, the renal vessels were bilaterally occluded with microvascular clamps. After 30 min, the clamps were removed for allowing recovery. Sham-operated mice underwent the same procedure, without renal vessel occlusion. Twenty-four hours after IR, these mice were euthanized, and blood and renal tissues were collected.

In the caspase-3 inhibitor experiment, Z-DEVD-FMK (500 ng/mice, MCE, Shanghai, China) was intraperitoneally injected daily for 4 days to inhibit caspase-3 in vivo (pretreatment of Z-DEVD-FMK for 1 day before cisplatin treatment plus 3-day injection of Z-DEVD-FMK after cisplatin treatment). After 72 h of cisplatin injection, the mice were euthanized, followed by the collection of blood and renal tissues. All the protocols were approved by Institutional Animal Care and Use Committee of Nanjing Medical University.

### Renal function and histology

Blood was collected into heparinized tubes and centrifuged at 3000 × *g* for 25 min to collect serum. Cr and BUN concentrations of the experimental mice were detected in the central laboratory of Nanjing Children’s Hospital using a serum biochemical autoanalyzer (Hitachi7600 modular chemistry analyzer, Hitachi Ltd., USA). Serum Cystatin C levels were analyzed using a mouse Cystatin C ELISA kit (Elascience, Wuhan, China), according to the manufacturer instructions. Kidney tissues were fixed with 4% paraformaldehyde for 24 h and then dehydrated in a graded ethanol series, cleared in xylene, and embedded in paraffin. PAS and HE staining was performed using standard methods. Tissue damage was scored by the percentage of renal tubules with cell lysis, loss of brush border, and cast formation (0, no damage; 1, ≤25%; 2, 26–50%; 3, 51–75%; 4, >75%).

### Immunohistochemical staining

For immunohistochemical staining, the following primary antibodies were used: anti-IL-6 (1:500; Abcam, MA, USA); anti-IL-1β (1:500; Abcam, MA, USA); anti-TNF-α (1:200; Abcam, MA, USA). The detailed protocol is mentioned in previous study^41^.

### Cell culture and treatments

The immortalized human tubular epithelial cell line (HK-2) was purchased from ATCC and was cultured in RPMI-1640 medium with 10% FBS (GIBCO, Brazil) at 37 °C with 5% CO_2_. According to the manufacturer’s instructions, plasmids with overexpressed GSDME-N or FL-GSDME, GSDME siRNA, or the corresponding negative control were transfected into cells using Lipofectamine 2000 (Invitrogen, Carlsbad, CA). To establish an in vitro AKI model, HK-2 cells were treated with cisplatin (10 μg/mL) for 24 h.

### Protein extraction and immunoblotting

The kidney sections were ground using a homogenizer or the cells were scratched and lysed in RIPA lysis buffer (Beyotime, Shanghai, China) with proteinase inhibitor (Roche, Switzerland) for 30 min on ice. Protein concentration was quantified using the Bicinchoninic acid assay (Beyotime, Shanghai, China). Protein samples (50 μg) were used to perform immunoblotting as described previously^42^. The specific primary antibodies used were: anti-GSDME (ab215191, Abcam, 1:1000), anti-caspase-3 (9662, CST, 1:1000), anti-cleaved caspase-3 (9661, CST, 1:1000), phospho-NF-κB p65 (3033, CST, 1:1000), NGAL (ab63929, Abcam, 1:1000), KIM-1 (AF1817, R&D systems, 1:1000), and anti-GAPDH (5174, CST, 1:10000). Peroxidase-conjugated goat anti-rabbit secondary antibodies (A0277, Beyotime, Shanghai, China) were used for immunoblotting.

### RNA extraction and quantitative real-time PCR

Total RNA of kidney tissues and cells was isolated using TRIzol Reagent (Invitrogen, Carlsbad, CA). Complementary DNA (cDNA) was synthesized using the PrimeScript RT Reagent Kit (TaKaRa, Tokyo, Japan). SYBR Green Premix Kit (Vazyme, Nanjing, China) was applied to perform the real-time PCR on QuantStudio 3 Real-time PCR System (Applied Biosystems, Foster City, CA, USA). The expression levels of genes were calculated using a comparative CT method, and GAPDH was used as an internal control. The sequences of the PCR primers in this study are listed in Table 1.

**Table 1:** Primer sequences for qRT-PCT.

Gene	Sequence
Mouse-KIM-1	F5′- ACATATCGTGGAATCACAACGAC-3′
	R5′- ACAAGCAGAAGATGGGCATTG-3′
Mouse-IL-1β	F5′-GCAACTGTTCCTGAACTCAACT-3′
	F5′-ATCTTTTGGGGTCCGTCAACT-3′
Mouse-IL-18	F5′-GACTCTTGCGTCAACTTCAAGG-3′
	F5′-CAGGCTGTCTTTTGTCAACGA-3′
Mouse-GAPDH	F5′-AGGTCGGTGTGAACGGATTTG-3′
	F5′-TGTAGACCATGTAGTTGAGGTCA-3′
Human-NGAL	F5′-CCACCTCAGACCTGATCCCA-3′
	F5′-CCCCTGGAATTGGTTGTCCTG-3′
Human-KIM-1	F5′-TGGCAGATTCTGTAGCTGGTT-3′
	F5′-AGAGAACATGAGCCTCTATTCCA-3′
Human-GAPDH	F5′-GGAGCGAGATCCCTCCAAAAT-3′
	F5′-GGCTGTTGTCATACTTCTCATGG-3′

### LDH assay

Cells were cultured in 96-well plates and then subjected to different treatments. One hour before assay, 20 μL of LDH release agent was added to the culture medium. LDH release levels were assessed by using LDH Cytotoxicity Assay Kit II (Beyotime, Shanghai, China), according to the manufacturer instructions.

### PI staining

The PI staining kit (Beyotime, Shanghai, China) was used to assess the pyroptotic cell death of cells. After co-incubation with Hoechst 33342 (5 μg/mL) for 10 min at 37 °C, the cells were digested and collected, followed by resuspension in PBS. Then, these cells were incubated with PI (10 μg/mL) at 4 °C for 10 min. After that, an inverted fluorescence microscope (Olympus, Tokyo, Japan) was used for photographing.

### ELISA

The kidney tissues ground using the homogenizer or the cells scratched were lysed in PBS. After centrifuging at 3000 × *g* for 25 min, the lysate was gathered. Mouse-IL-1β enzyme-linked immunosorbent assay (R&D, MN, USA) was used to measure IL-1β concentration in kidney tissues. After collecting the cell culture supernatants, IL-1β and IL-6 levels were measured by using the human IL-1β kit (R&D, MN, USA) and human IL-6 kit (Dakewe, Beijing, China). The absorbance value at 450 nm was measured.

### Cell viability assay

Cells were seeded in the 96-well culture plates and then subjected to the indicated treatments. Cell viability was measured using the CCK-8 assay kit (KeyGen, Nanjing China), according to the manufacturer’s protocol.

### Microscopic imaging

Cells were first seeded in the confocal culture dishes and were subjected to transfections or cisplatin treatment when reaching a confluency of 80%. Static bright-field images were captured using Olympus IX71 microscope (Tokyo, Japan). To examine the morphology of pyroptotic cells, transmission electron microscopy S3400N-II (Hitachi, Japan) was used.

### Caspase-1 activity assay

Caspase-1 activity of HK-2 cells was measured by using a Caspase-1 Activity Assay Kit (Beyotime, C1102, China) as instructed by the manufacture. One unit of caspase-1 activity is the amount of enzyme that will cleave 1.0 nmol of the colorimetric substrate Ac-YVAD-pNA per hour at 37 °C under saturated substrate concentrations. The protein concentration of HK-2 cells was detected by using Bradford Protein Assay Kit (Beyotime, P0006, China) following the instructions of manufacture.

### TUNEL assay

Apoptosis of renal cells was determined by using a TUNEL Bright Green Apoptosis Detection Kit (Vazyme, A112-01/02/03, China) following the protocol provided by the manufacture. The images of TUNEL-positive signals were acquired with a laser scanning confocal microscopy (CarlZeiss LSM710). The number of apoptotic cells for each sample was counted for quantification.

### Statistical analyses

Results were presented as mean ± SEM and were analyzed with GraphPad Prism software using analysis of variance followed by Student’s *t*-test. *P* < 0.05 was considered statistically significant.

## Supplementary information

Supplemental Figure S1

Supplemental Figure S2

Supplemental Figure S3

Supplemental Figure S4

Supplemental Figure S5

Supplementary figure legends-S1-S5

Original Western blots
