# Supplementary figures and images for: Gasdermin E deficiency attenuates acute kidney injury by inhibiting pyroptosis and inflammation
Source: Cell Death Dis. 2021 Feb 1;12(2):139. doi: 10.1038/s41419-021-03431-2 (PMC7862699; doi:10.1038/s41419-021-03431-2)

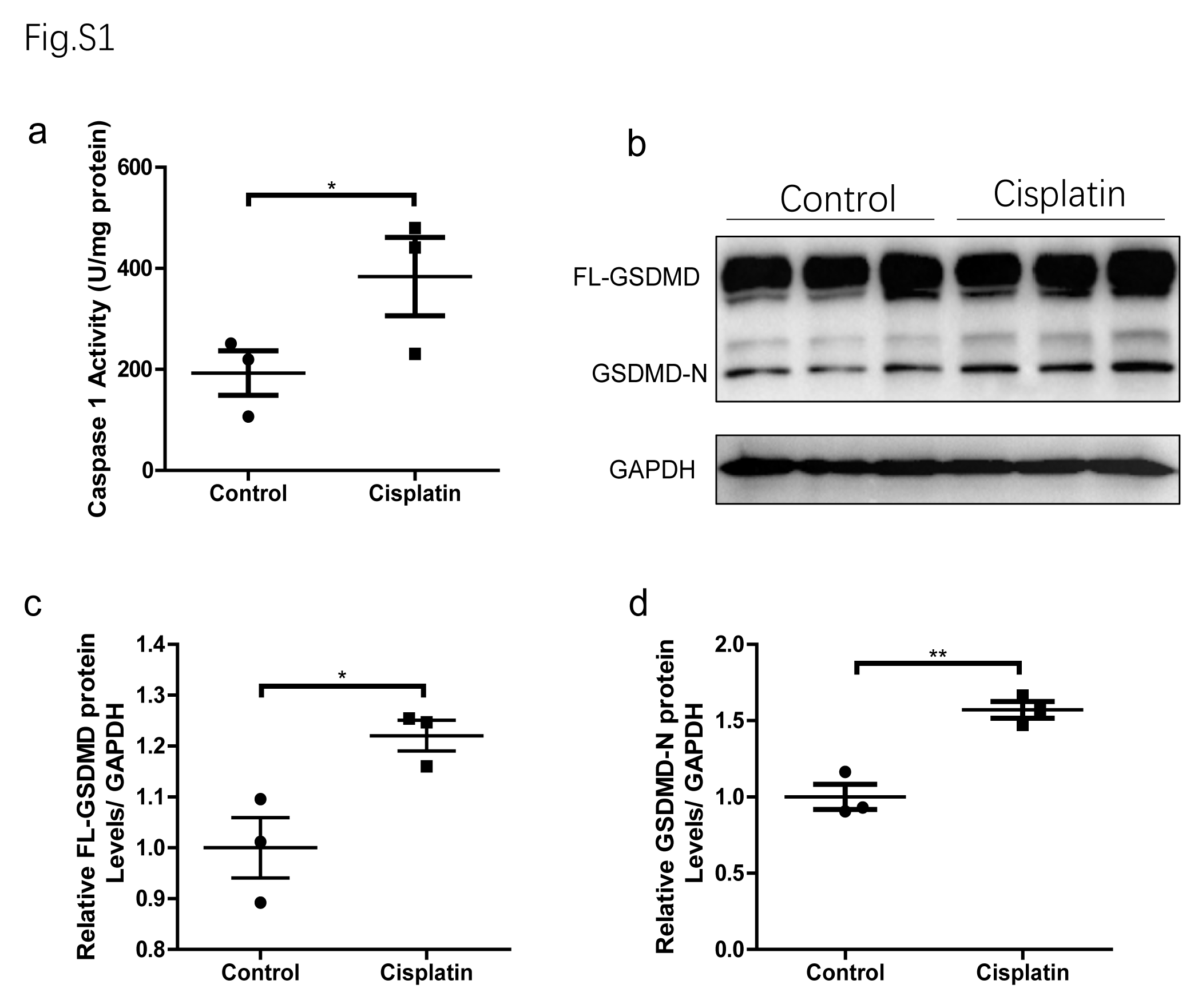

Supplement: Supplementary file 1 — Supplemental Figure S1 [file 41419_2021_3431_MOESM1_ESM.tif]

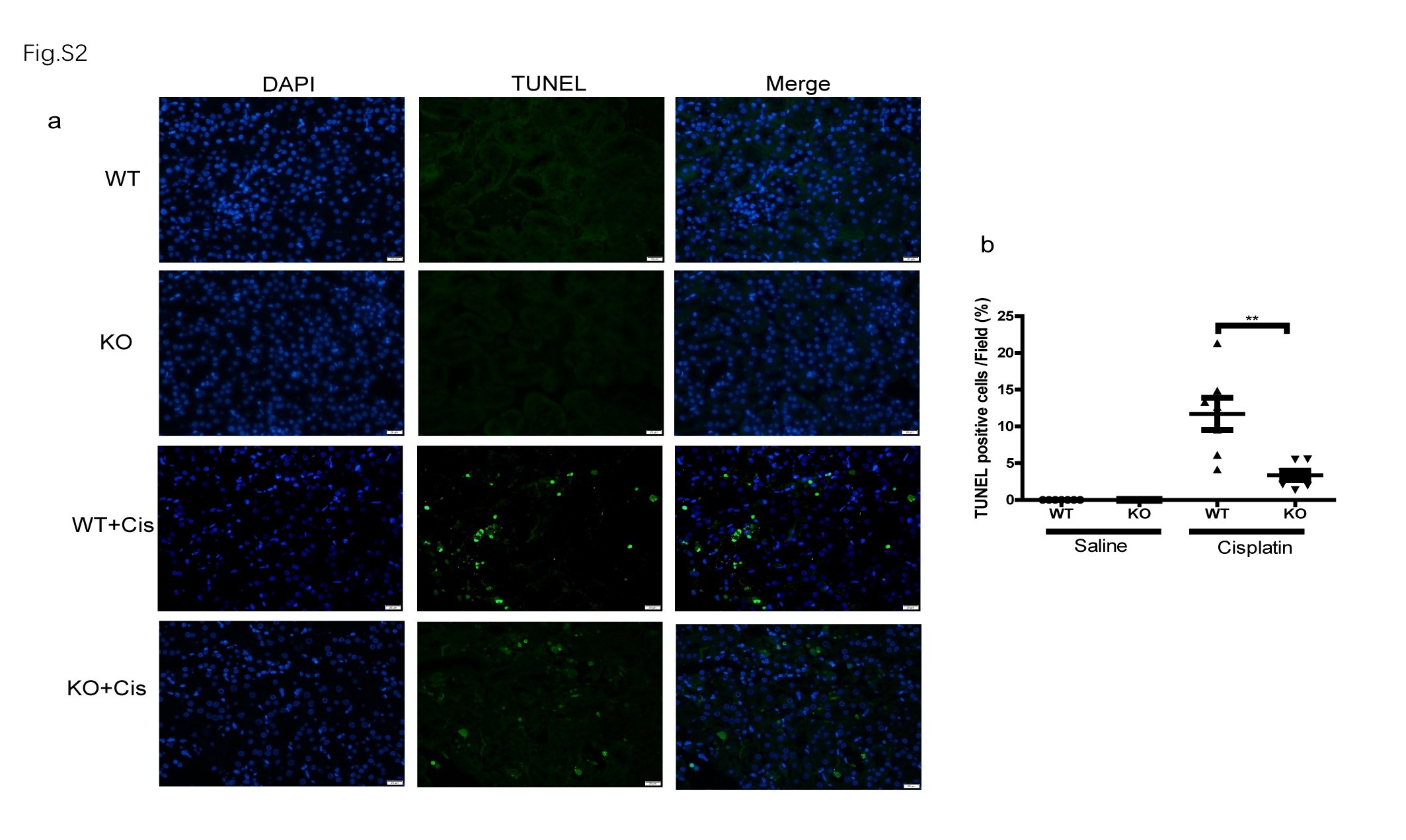

Supplement: Supplementary file 2 — Supplemental Figure S2 [file 41419_2021_3431_MOESM2_ESM.tif]

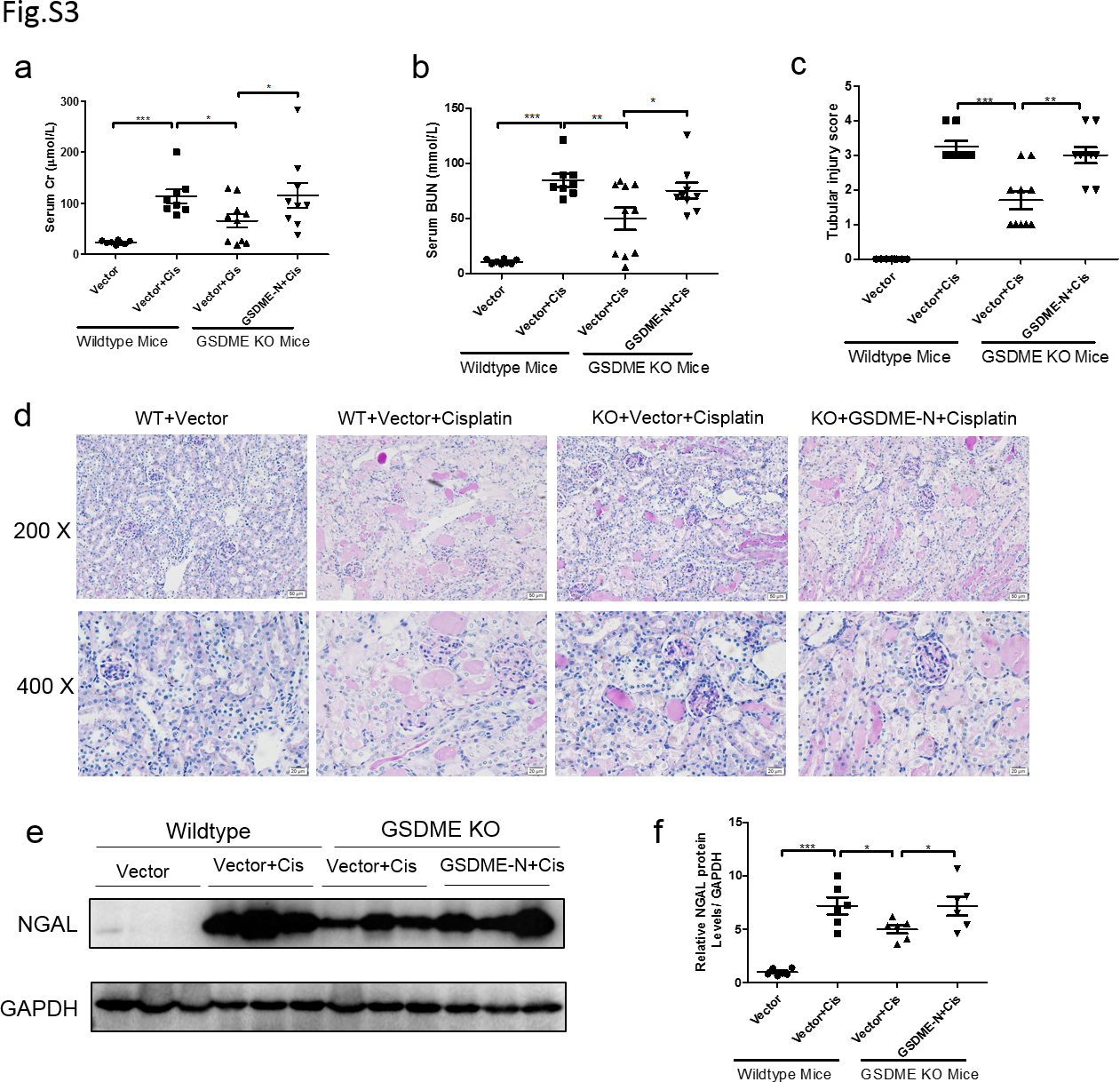

Supplement: Supplementary file 3 — Supplemental Figure S3 [file 41419_2021_3431_MOESM3_ESM.tif]

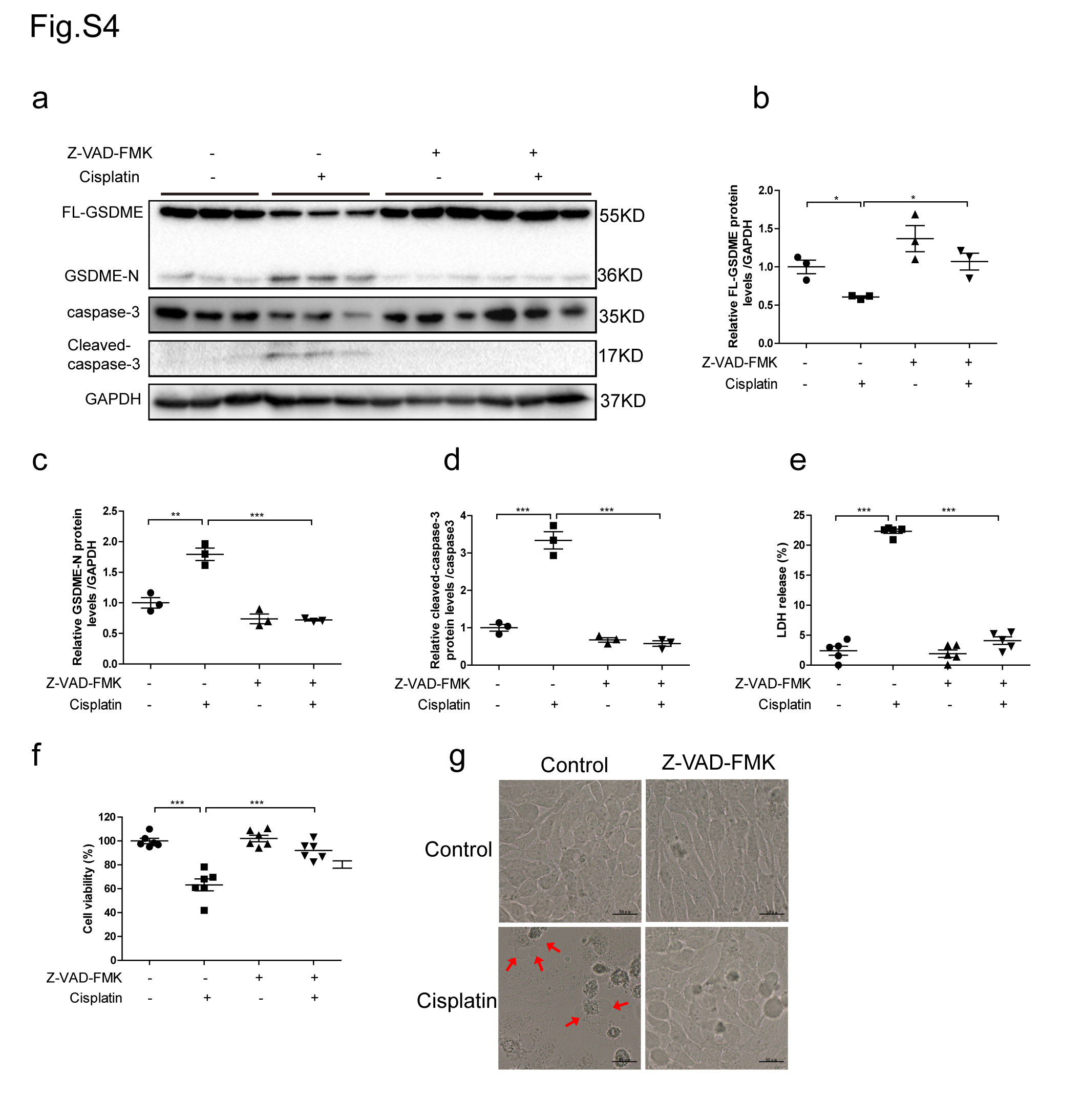

Supplement: Supplementary file 4 — Supplemental Figure S4 [file 41419_2021_3431_MOESM4_ESM.tif]

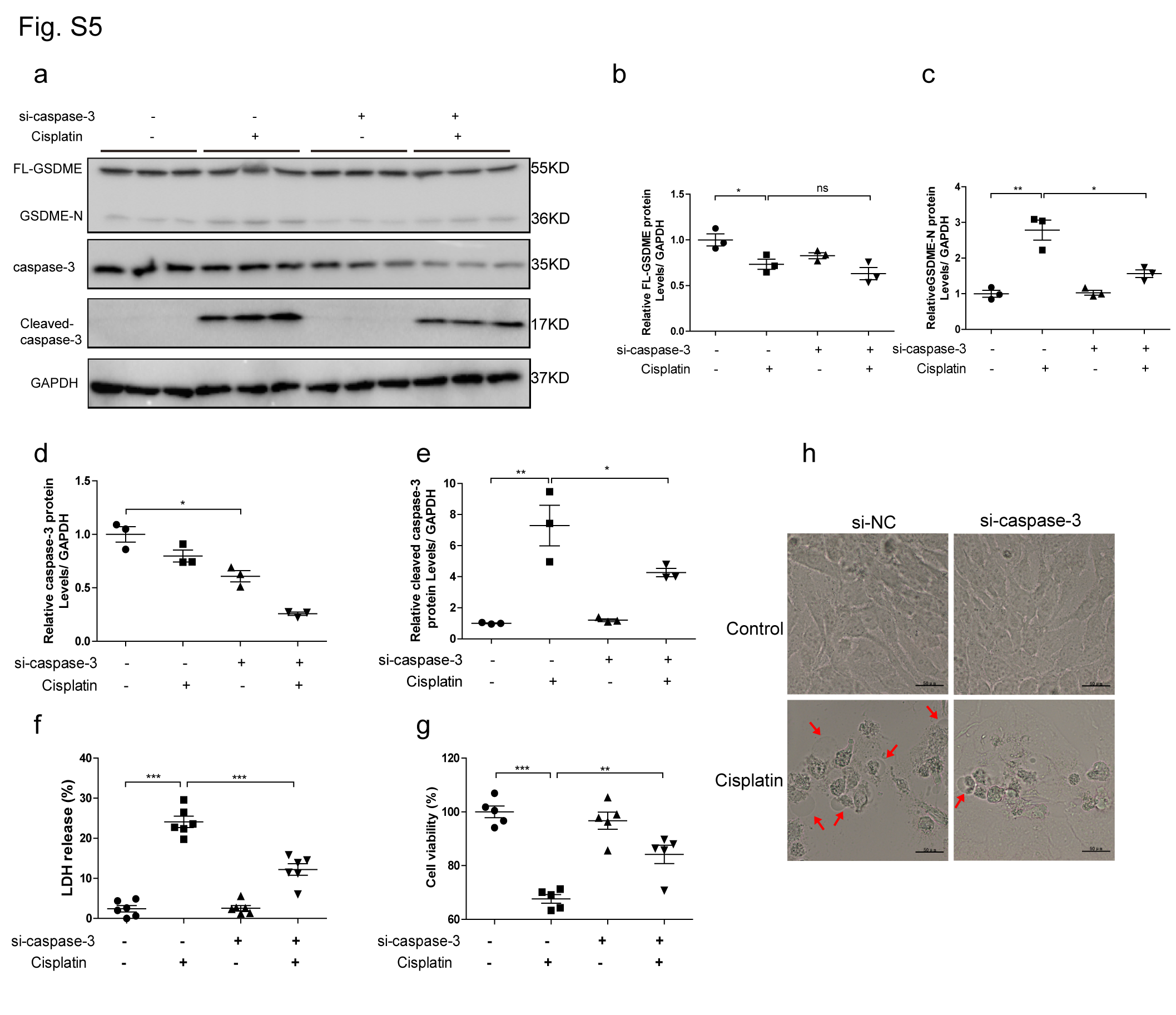

Supplement: Supplementary file 5 — Supplemental Figure S5 [file 41419_2021_3431_MOESM5_ESM.tif]
