# Supplementary material for: Gasdermin E deficiency attenuates acute kidney injury by inhibiting pyroptosis and inflammation
Source: Cell Death Dis. 2021 Feb 1;12(2):139. doi: 10.1038/s41419-021-03431-2 (PMC7862699; doi:10.1038/s41419-021-03431-2)
Supplement: Supplementary file 6 — Supplementary figure legends-S1-S5 [file 41419_2021_3431_MOESM6_ESM.docx]

**Supplementary Figure Legends**

**Fig. S1 Cisplatin activated caspase 1 and induced the cleavage of GSDMD in human renal epithelial cells**

Human renal epithelial cells (HK-2) subjected to cisplatin (10 μg/ml) were used to determine the activity of caspase-1 and the cleavage of GSDMD. (a) The activity of caspase 1 in HK-2 cells subjected to cisplatin (n=3). (b) Protein level of GSDMD in HK-2 cells with cisplatin treatment (n=3). *P<0.05.

**Fig. S2 GSDME deletion alleviated cisplatin-induced apoptosis in kidney**

(a) Representative images of TUNEL staining (magniﬁcation × 400, scale bar: 20 μm ) (b)Quantification of TUNEL positive cells in mice kidneys.

**Fig. S3 GSDME-N overexpression abolished the protection against cisplatin-induced acute kidney injury in GSDME deletion mice**

WT and KO mice (10-12 weeks) were subjected to 80 μg control vector (pEGFP-N1) or GSDME-N (pEGFP-N1-GSDME-N) plasmids via tail vein injection followed by 25mg/kg of cisplatin or the same volume of saline 36 hours later. (a&b) Serum Cr and BUN levels of mice (n=8-10). (c)The associated renal tubular injury score analysis. (d) Representative image of PAS staining (magnification ×200, scale bar=50 μm; magnification ×400, scale bar=20 μm). (f) Renal protein level of NGAL in kidney tissues detected by immunoblotting. (g) Quantitative analysis of the Western blots of NGAL in kidney tissues. *P<0.05, **P<0.01, and ***P<0.001.

**Fig. S4 Caspase inhibitor Z-VAD-FMK** **relieved cisplatin-induced cell injury and pyroptosis in human TECs**

Human TECs (HK-2) were incubated with Z-VAD-FMK for 3 h and subsequently treated with cisplatin (10μg/mL) for 24 h. (a-d) Effect of regulation of Z-VAD-FMK on FL-GSDME, GSDME-N, caspase-3, and cleaved caspase-3 protein levels (n=3). (e) LDH release of cells treated with Z-VAD-FMK (n=5). (f) Effect of Z-VAD-FMK on cell viability (n=6). (g) Representative bright-ﬁeld microscopic images (scale bar=50 μm) of cells. Red arrowheads indicate large bubbles from the plasma membrane. *P<0.05, **P<0.01, and ***P<0.001.

**Fig. S5 Knock-down of caspase-3 alleviated cisplatin-induced cell injury and pyroptosis in human TECs**

Human TECs (HK-2) were transfected with caspase-3 siRNA or the negative control and subsequently treated with cisplatin (10μg/mL) for 24 h. (a-e) Protein levels of FL-GSDME, GSDME-N, caspase-3, and cleaved caspase-3 detected by immunoblotting (n=3). (f) LDH release in different groups of cells (n=6). (g) Cell viability of different groups of cells (n=5). (h) Representative bright-ﬁeld microscopic images (scale bar=50 μm) of cells. Red arrowheads indicate large bubbles from the plasma membrane. *P<0.05, **P<0.01, and ***P<0.001.
