# Supplementary material for: Gasdermin E deficiency attenuates acute kidney injury by inhibiting pyroptosis and inflammation
Source: Cell Death Dis. 2021 Feb 1;12(2):139. doi: 10.1038/s41419-021-03431-2 (PMC7862699; doi:10.1038/s41419-021-03431-2)
Supplement: Supplementary file 7 — Original Western blots [file 41419_2021_3431_MOESM7_ESM.pptx]

## Slide 1
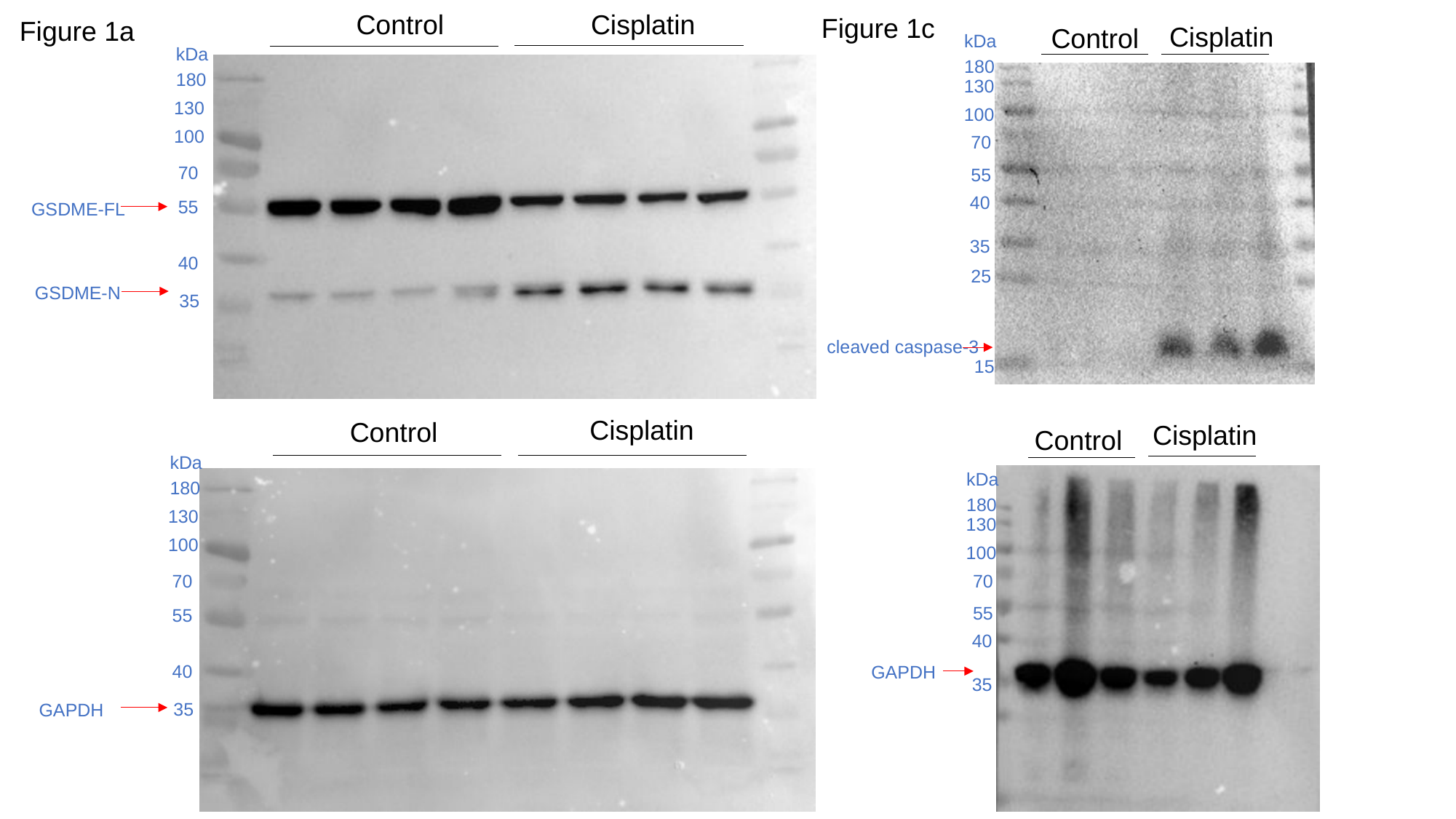

Cisplatin
Control
Figure 1c
Figure 1a
Cisplatin
Control
kDa
180
130
100
70
55
40
35
25
15
kDa
180
130
100
70
55
40
35
GSDME-FL
GSDME-N
cleaved caspase-3
Cisplatin
Control
Cisplatin
Control
kDa
180
130
100
70
55
40
35
kDa
180
130
100
70
55
40
35
GAPDH
GAPDH

## Slide 2
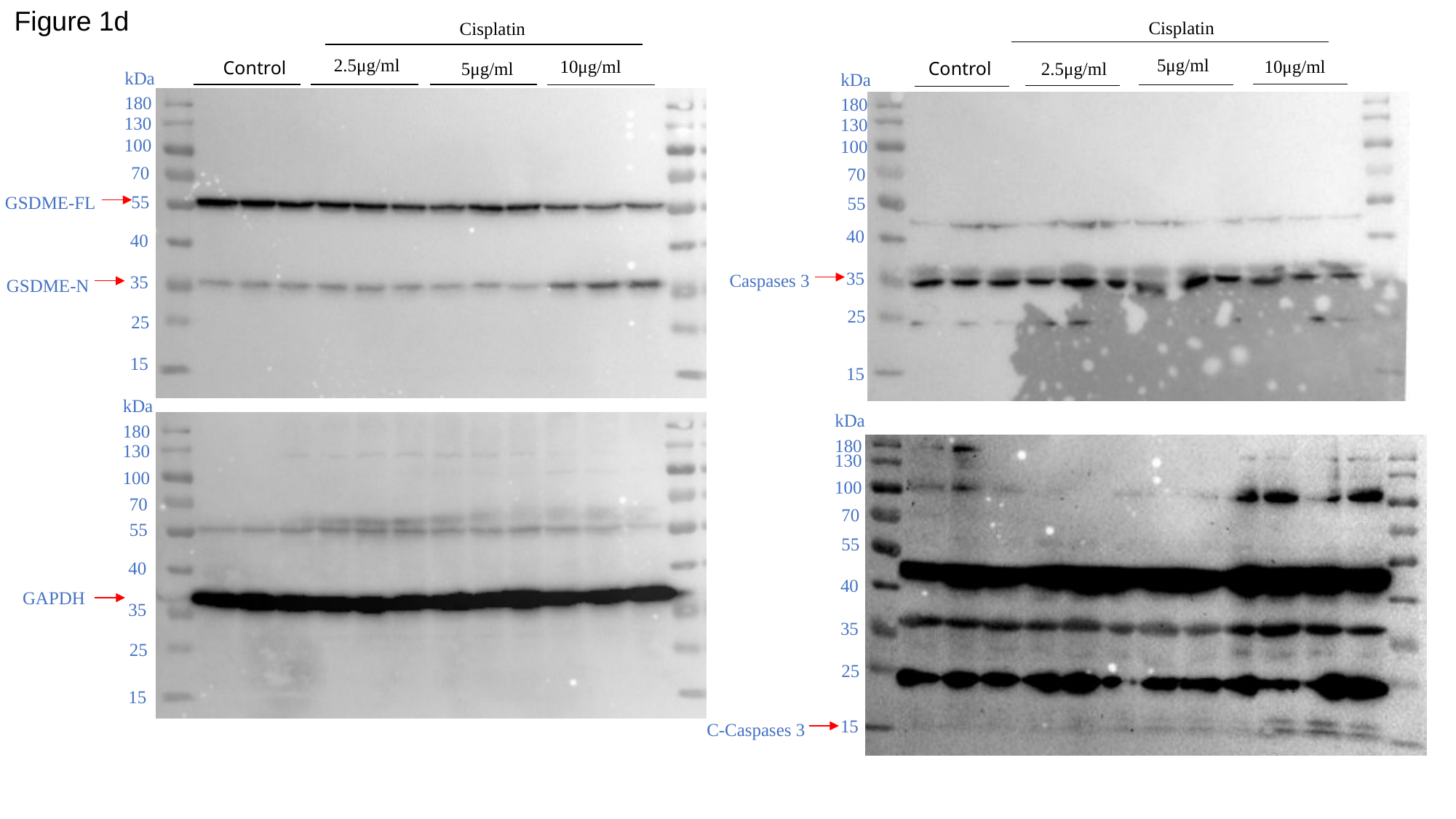

Figure 1d
Cisplatin
Cisplatin
5μg/ml
2.5μg/ml
 10μg/ml
 10μg/ml
Control
Control
5μg/ml
2.5μg/ml
kDa
180
130
100
70
55
40
35
25
15
kDa
180
130
100
70
55
40
35
25
15
GSDME-FL
Caspases 3
GSDME-N
kDa
180
130
100
70
55
40
35
25
15
kDa
180
130
100
70
55
40
35
25
15
GAPDH
C-Caspases 3

## Slide 3
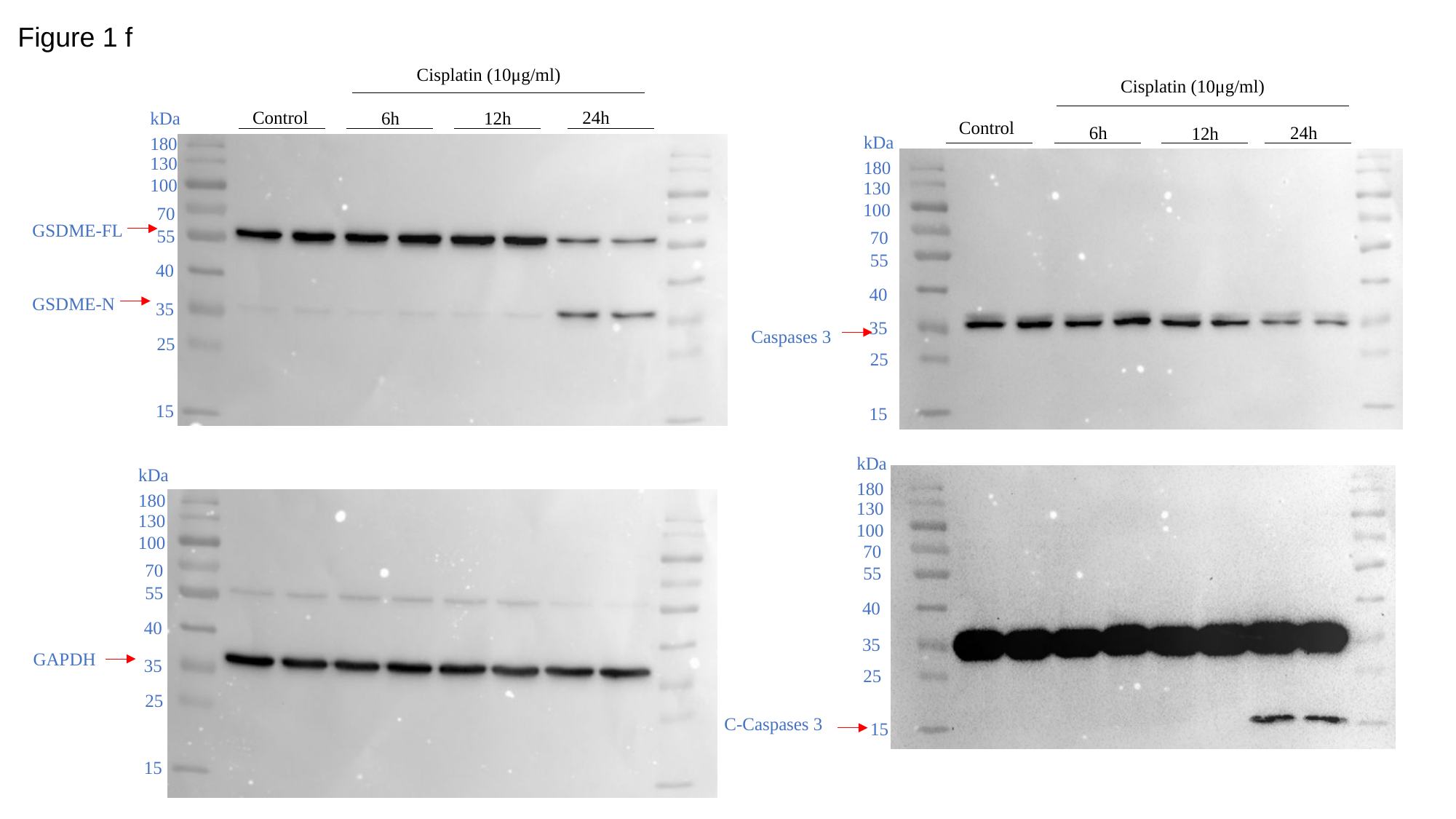

Figure 1 f
Cisplatin (10μg/ml)
Cisplatin (10μg/ml)
24h
 Control
kDa
180
130
100
70
55
40
35
25
15
6h
 12h
 Control
24h
6h
 12h
kDa
180
130
100
70
55
40
35
25
15
GSDME-FL
GSDME-N
Caspases 3
kDa
180
130
100
70
55
40
35
25
15
kDa
180
130
100
70
55
40
35
25
15
GAPDH
C-Caspases 3

## Slide 4
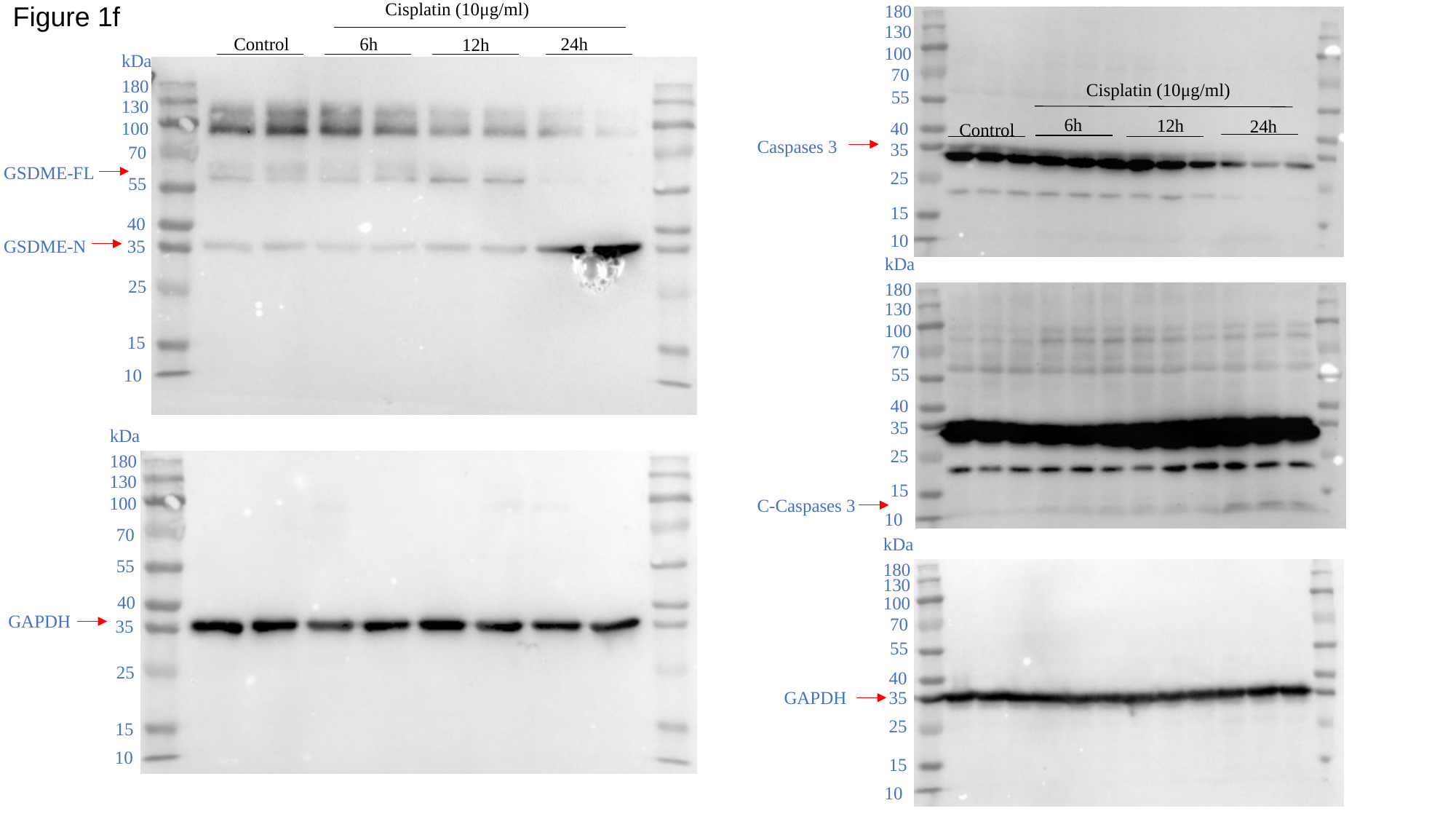

kDa
180
130
100
70
55
40
35
25
15
Cisplatin (10μg/ml)
Figure 1f
24h
 Control
6h
 12h
kDa
180
130
100
70
55
40
35
25
15
Cisplatin (10μg/ml)
6h
 12h
24h
 Control
Caspases 3
GSDME-FL
10
GSDME-N
kDa
180
130
100
70
55
40
35
25
15
10
kDa
180
130
100
70
55
40
35
25
15
C-Caspases 3
10
kDa
180
130
100
70
55
40
35
25
15
GAPDH
GAPDH
10
10

## Slide 5
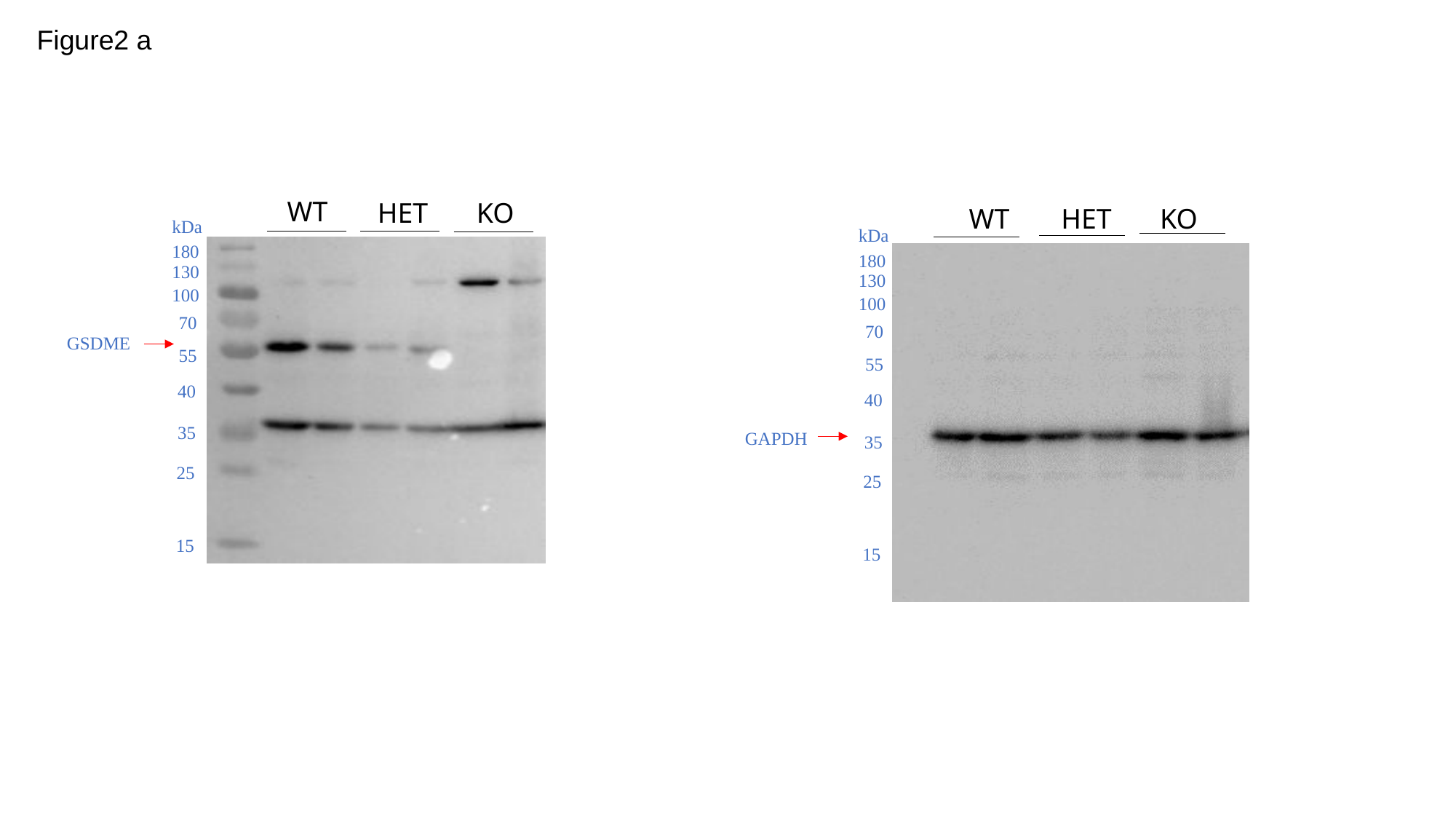

Figure2 a
WT
HET
KO
HET
KO
WT
kDa
180
130
100
70
55
40
35
25
15
kDa
180
130
100
70
55
40
35
25
15
GSDME
GAPDH

## Slide 6
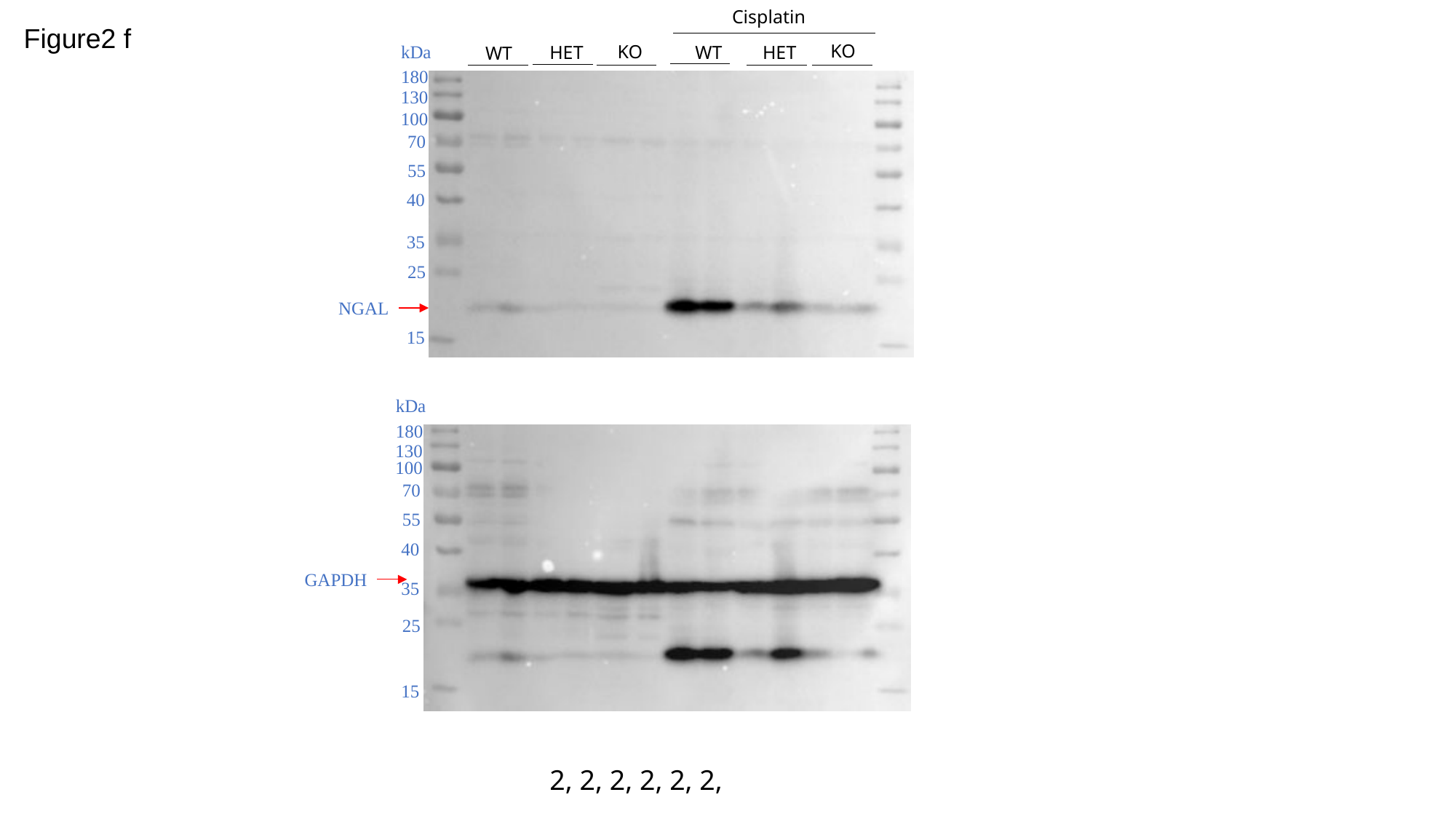

Cisplatin
Figure2 f
KO
KO
kDa
180
130
100
70
55
40
35
25
15
HET
HET
WT
WT
NGAL
kDa
180
130
100
70
55
40
35
25
15
GAPDH
2, 2, 2, 2, 2, 2,

## Slide 7
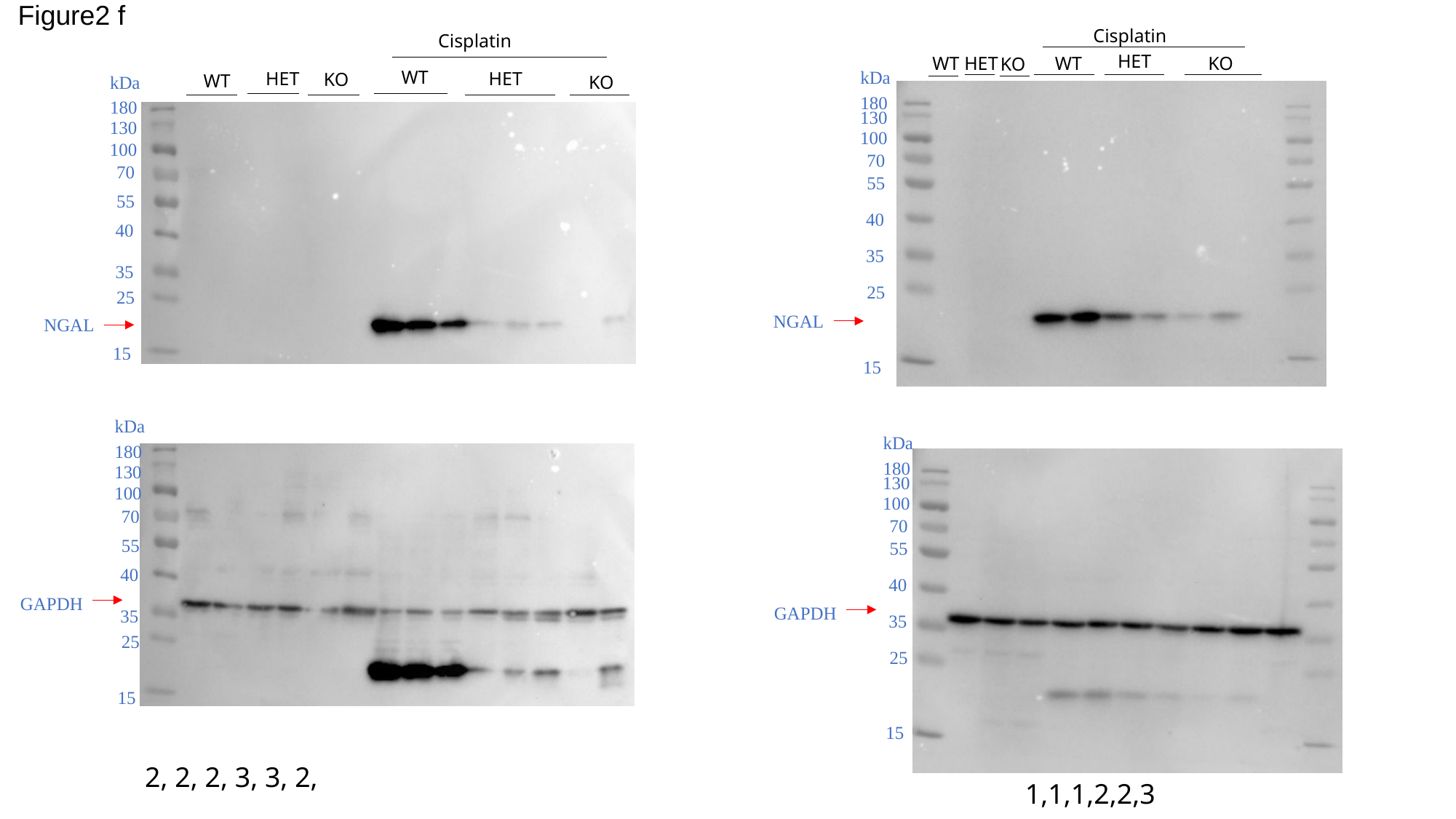

Figure2 f
Cisplatin
Cisplatin
HET
KO
WT
WT
HET
KO
kDa
180
130
100
70
55
40
35
25
15
WT
HET
HET
KO
WT
kDa
180
130
100
70
55
40
35
25
15
KO
NGAL
NGAL
kDa
180
130
100
70
55
40
35
25
15
kDa
180
130
100
70
55
40
35
25
15
GAPDH
GAPDH
2, 2, 2, 3, 3, 2,
1,1,1,2,2,3

## Slide 8
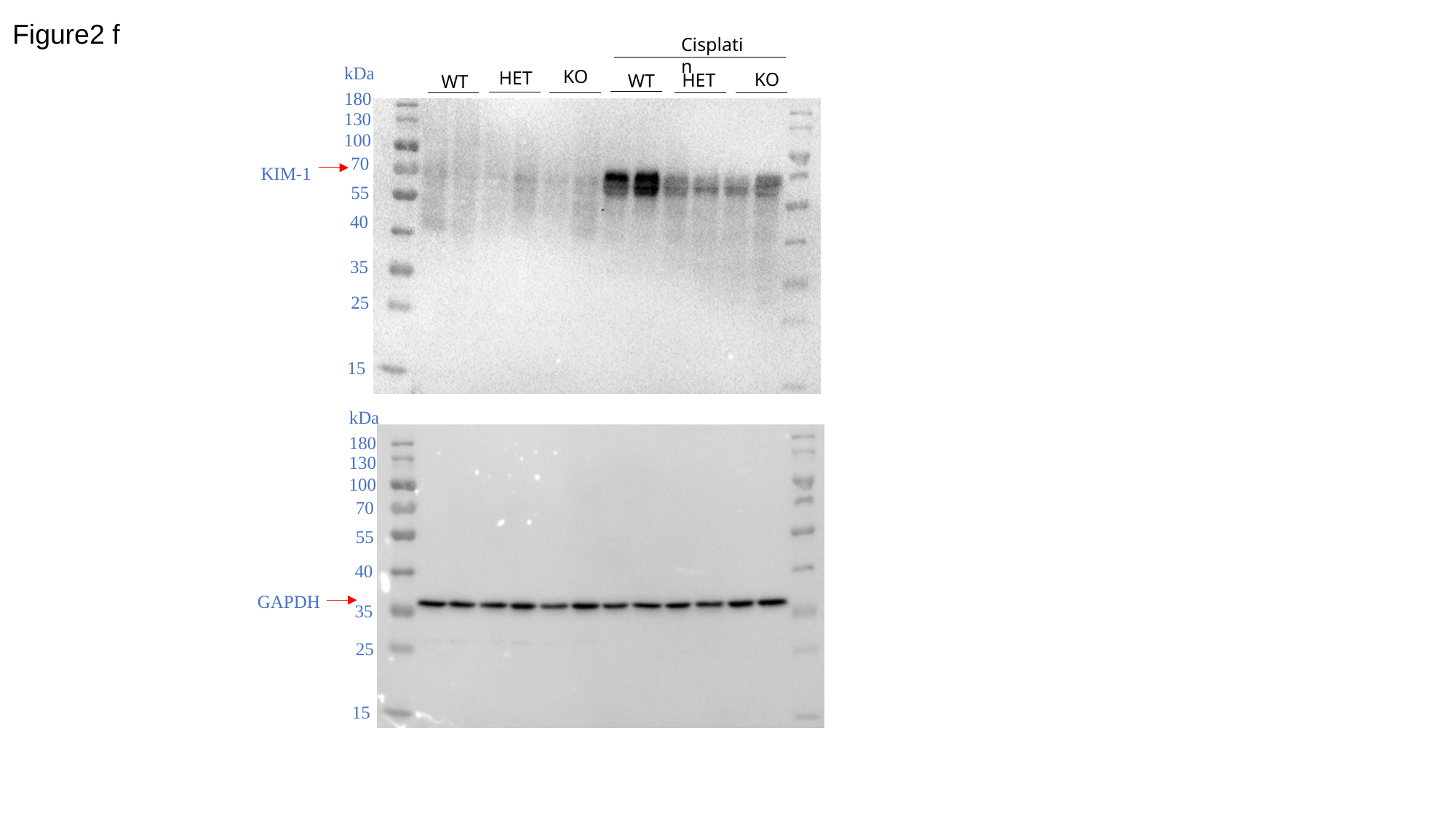

Figure2 f
Cisplatin
kDa
180
130
100
70
55
40
35
25
15
KO
HET
KO
HET
WT
WT
KIM-1
kDa
180
130
100
70
55
40
35
25
15
GAPDH

## Slide 9
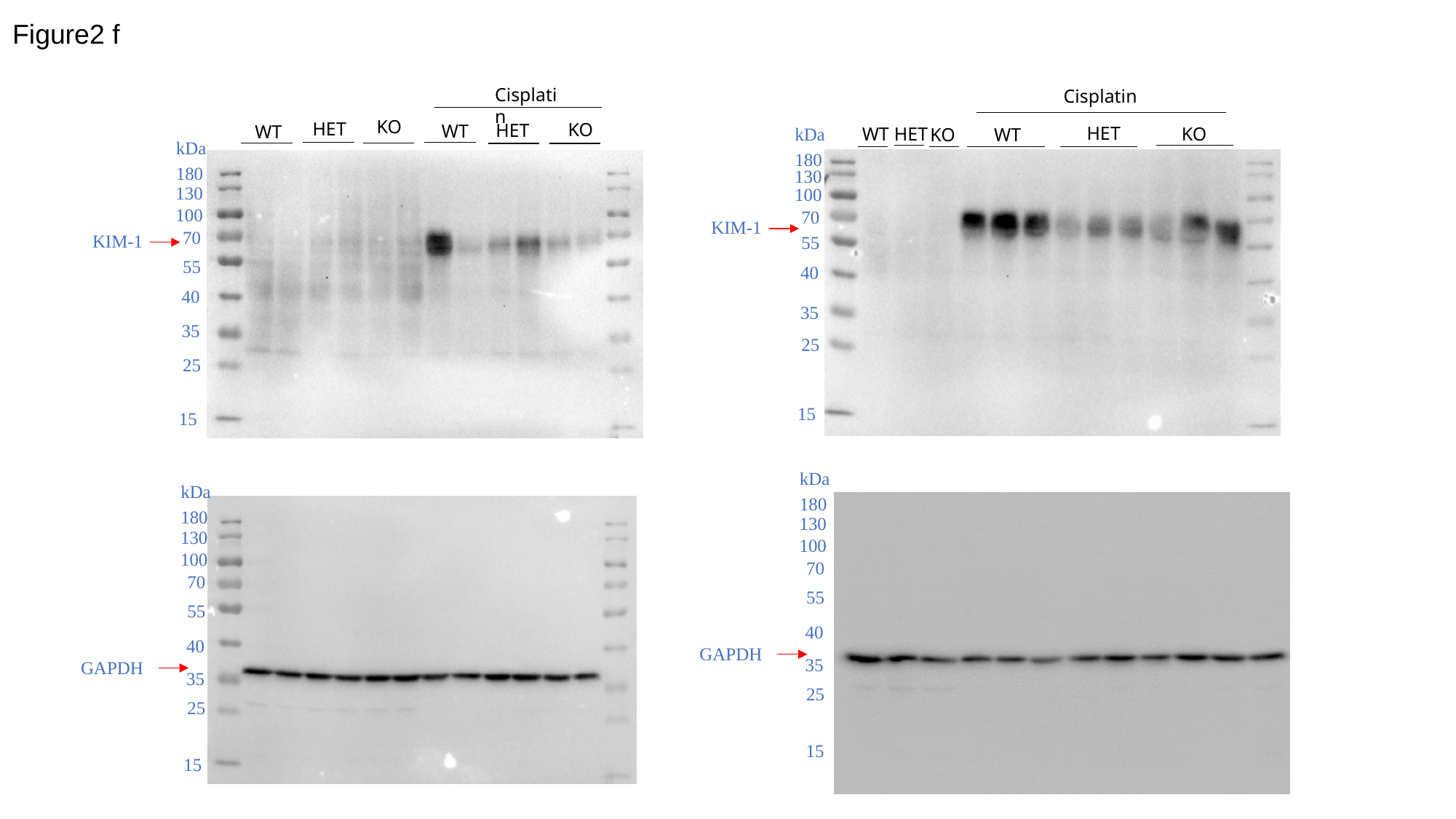

Figure2 f
Cisplatin
Cisplatin
KO
HET
KO
HET
WT
WT
HET
KO
kDa
180
130
100
70
55
40
35
25
15
WT
HET
KO
WT
kDa
180
130
100
70
55
40
35
25
15
KIM-1
KIM-1
kDa
180
130
100
70
55
40
35
25
15
kDa
180
130
100
70
55
40
35
25
15
GAPDH
GAPDH

## Slide 10
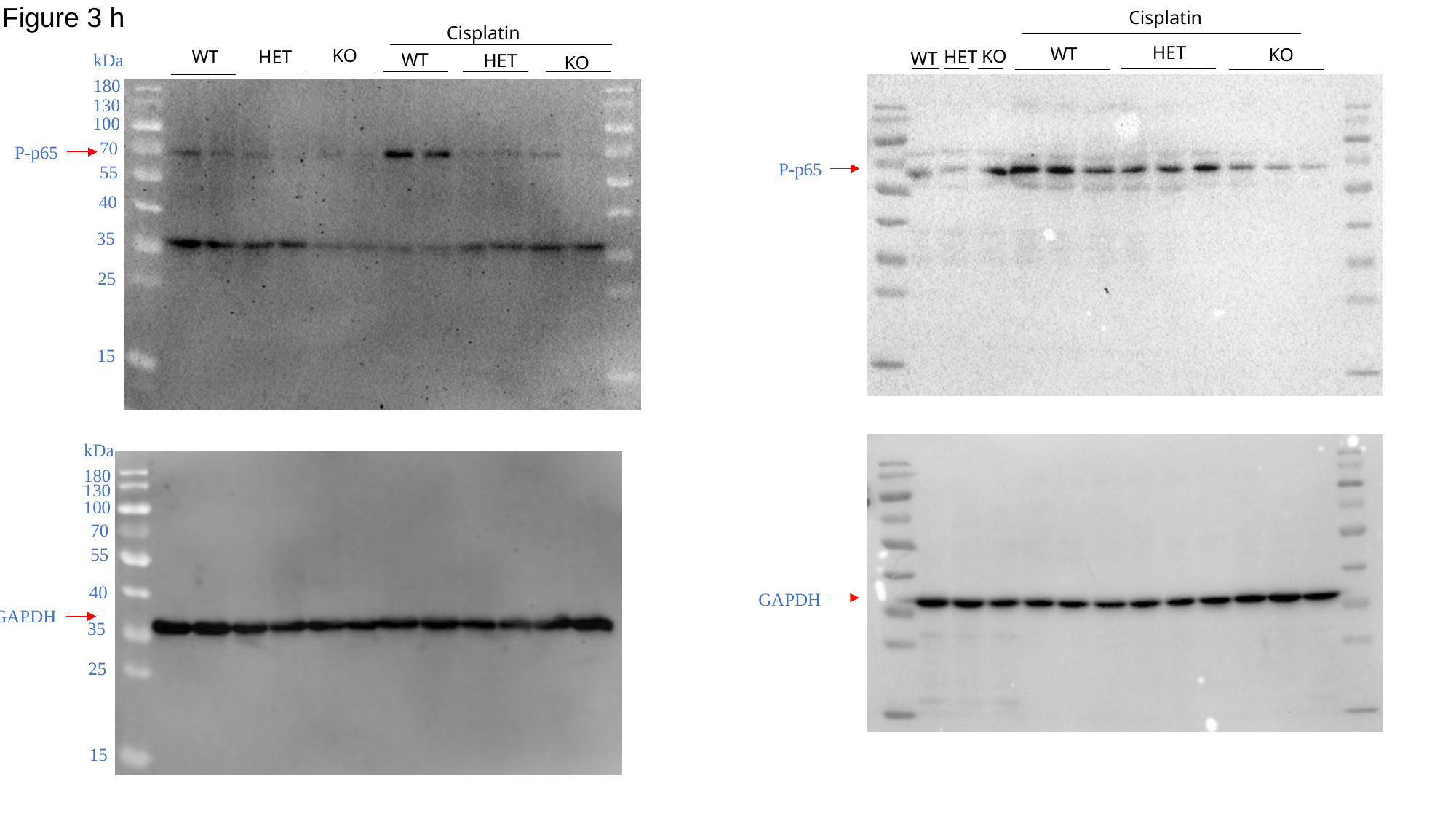

Figure 3 h
Cisplatin
Cisplatin
HET
WT
KO
KO
KO
HET
HET
WT
WT
kDa
180
130
100
70
55
40
35
25
15
WT
HET
KO
P-p65
P-p65
kDa
180
130
100
70
55
40
35
25
15
GAPDH
GAPDH

## Slide 11
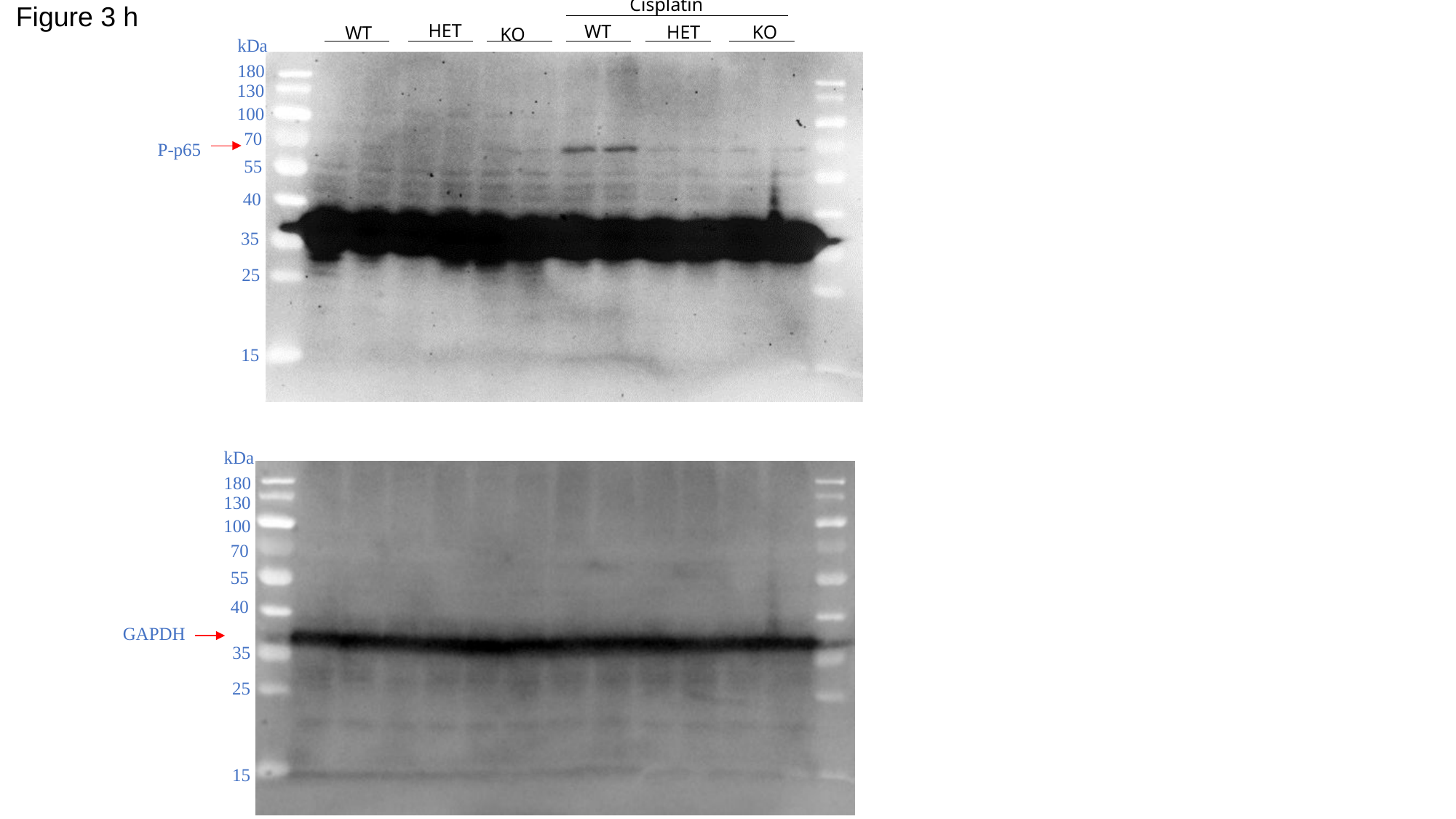

Cisplatin
Figure 3 h
HET
WT
KO
HET
WT
KO
kDa
180
130
100
70
55
40
35
25
15
P-p65
kDa
180
130
100
70
55
40
35
25
15
GAPDH

## Slide 12
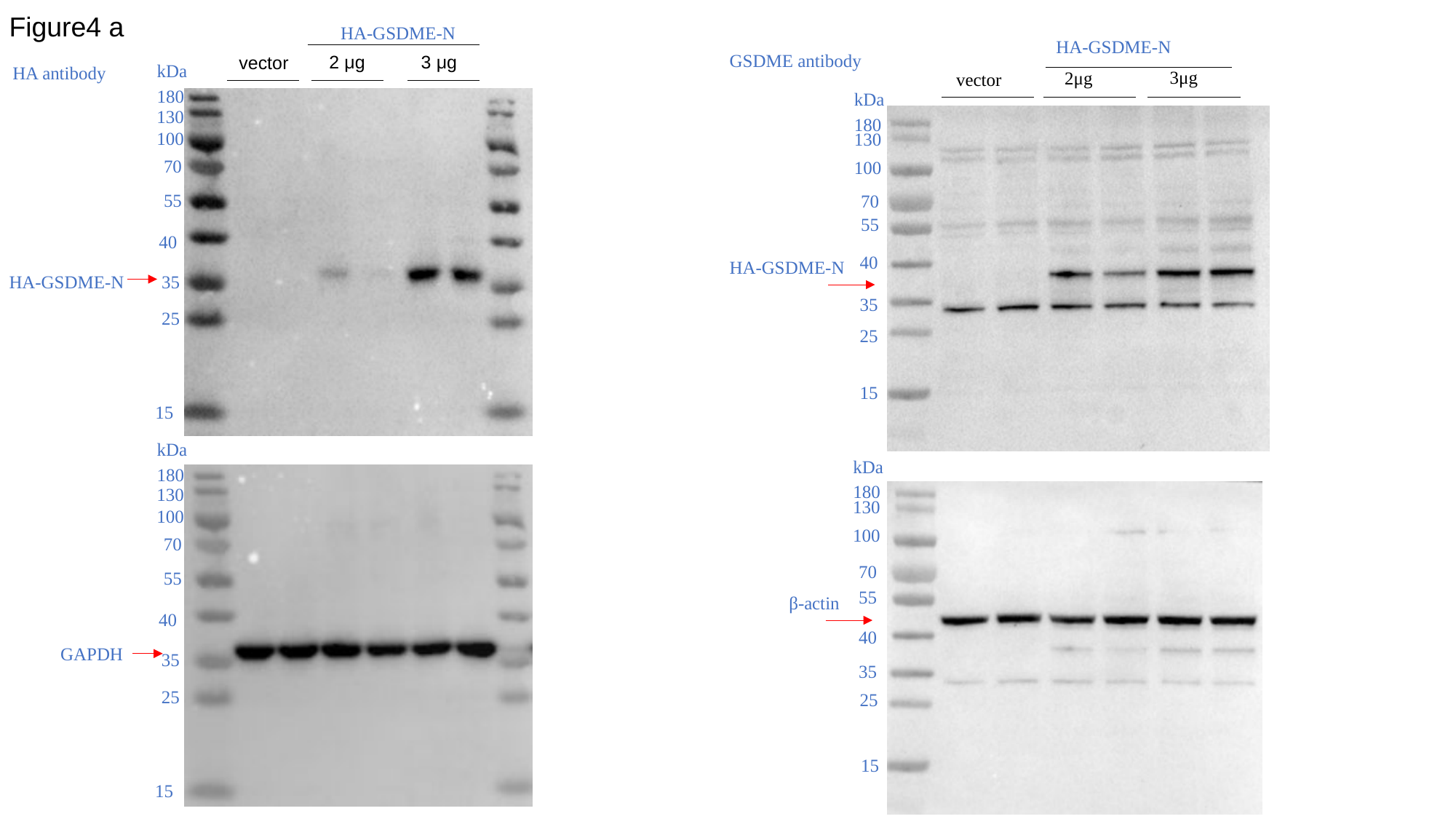

Figure4 a
HA-GSDME-N
HA-GSDME-N
GSDME antibody
2 μg
3 μg
vector
kDa
180
130
100
70
55
40
35
25
15
HA antibody
3μg
2μg
vector
kDa
180
130
100
70
55
40
35
25
15
HA-GSDME-N
HA-GSDME-N
kDa
180
130
100
70
55
40
35
25
15
kDa
180
130
100
70
55
40
35
25
15
β-actin
GAPDH

## Slide 13
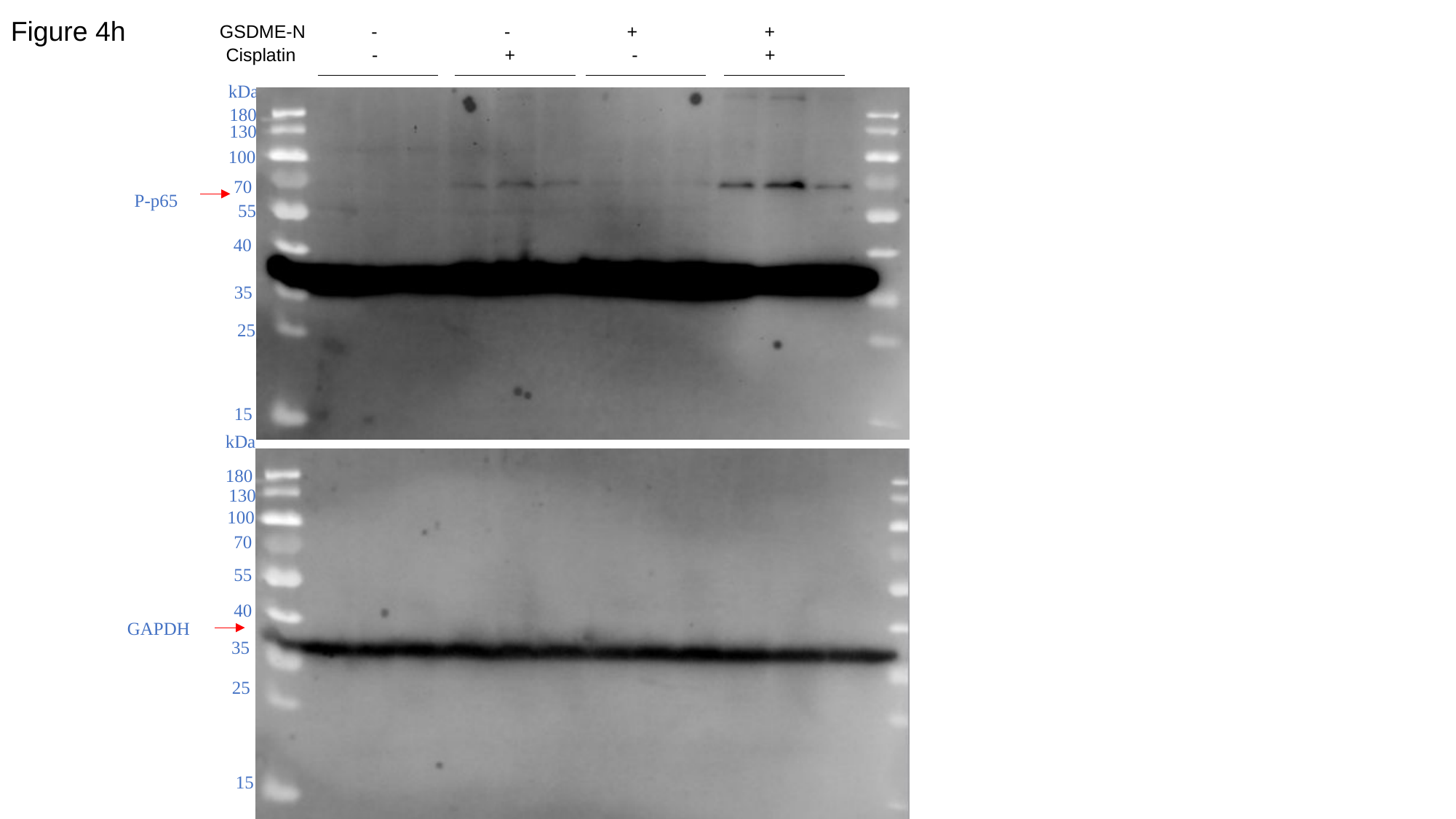

Figure 4h
GSDME-N - - + +
Cisplatin - + - +
kDa
130
100
70
55
40
35
25
15
180
P-p65
kDa
180
130
100
70
55
40
35
25
15
GAPDH

## Slide 14
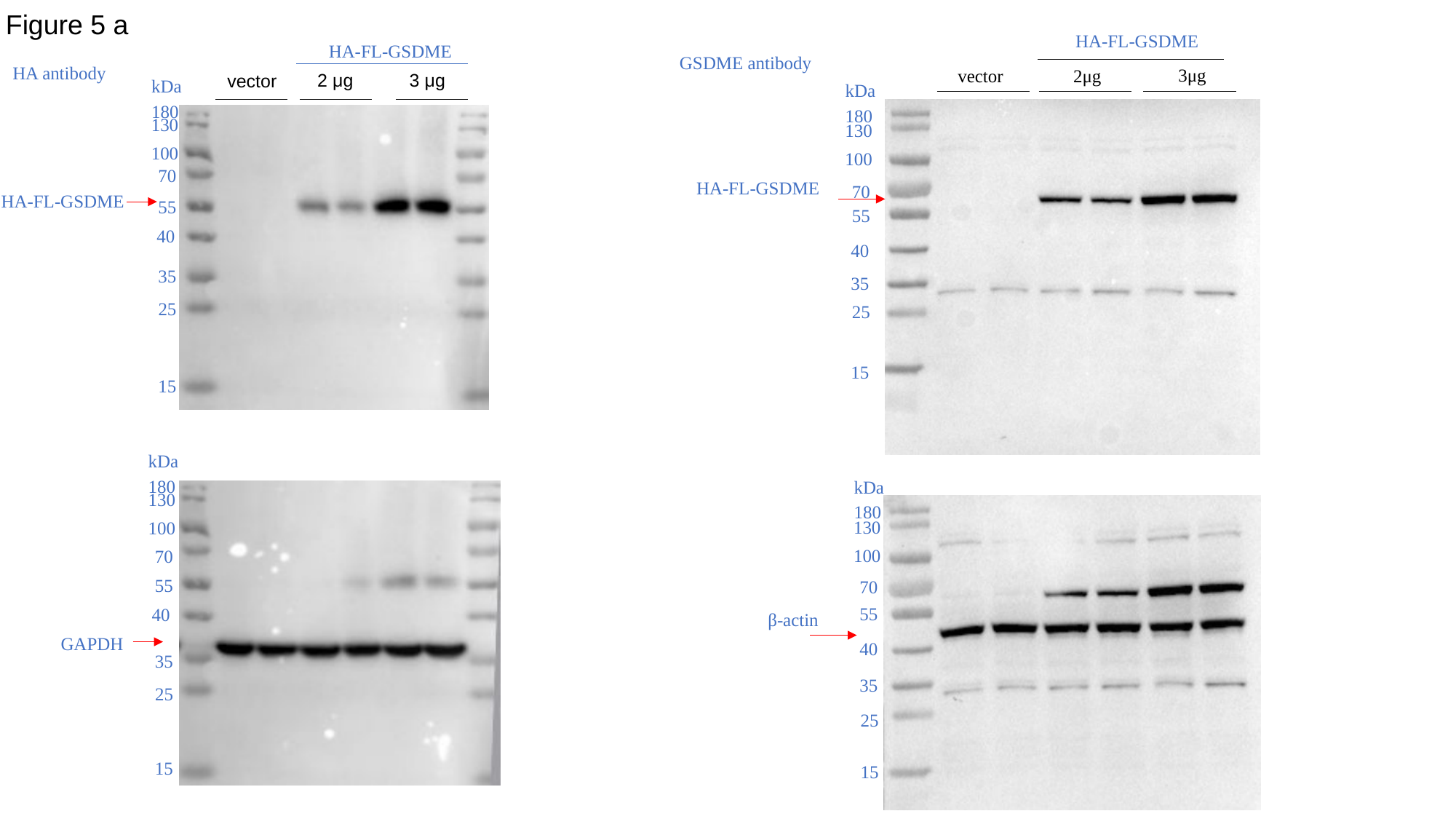

Figure 5 a
HA-FL-GSDME
HA-FL-GSDME
GSDME antibody
HA antibody
3μg
vector
2μg
2 μg
3 μg
vector
kDa
180
130
100
70
55
40
35
25
15
kDa
180
130
100
70
55
40
35
25
15
HA-FL-GSDME
HA-FL-GSDME
kDa
180
130
100
70
55
40
35
25
15
kDa
180
130
100
70
55
40
35
25
15
β-actin
GAPDH

## Slide 15
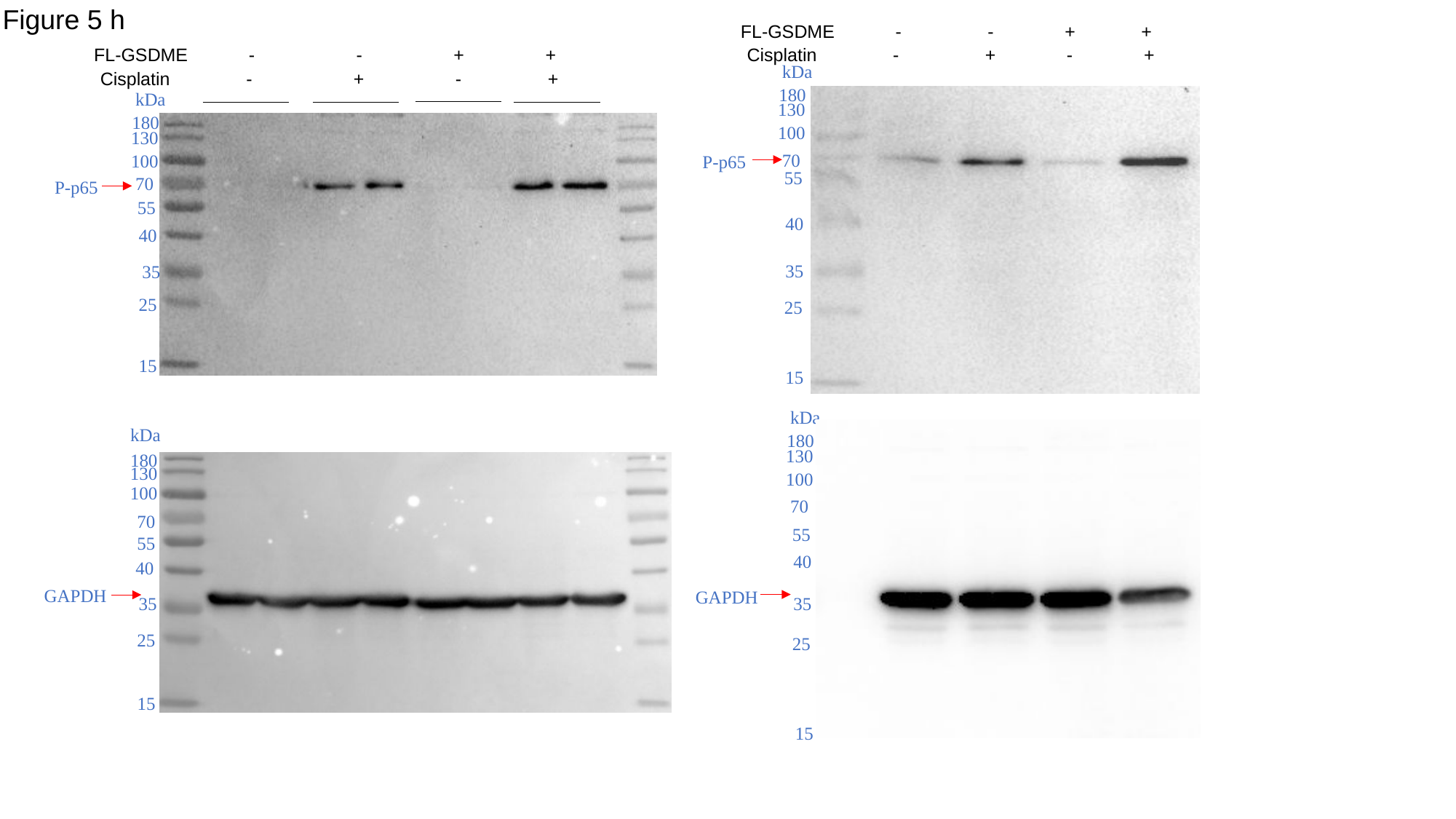

Figure 5 h
FL-GSDME - - + +
Cisplatin - + - +
FL-GSDME - - + +
kDa
130
100
70
55
40
35
25
15
180
Cisplatin - + - +
kDa
130
100
70
55
40
35
25
15
180
P-p65
P-p65
kDa
130
100
70
55
40
35
25
15
180
kDa
180
130
100
70
55
40
35
25
15
GAPDH
GAPDH

## Slide 16
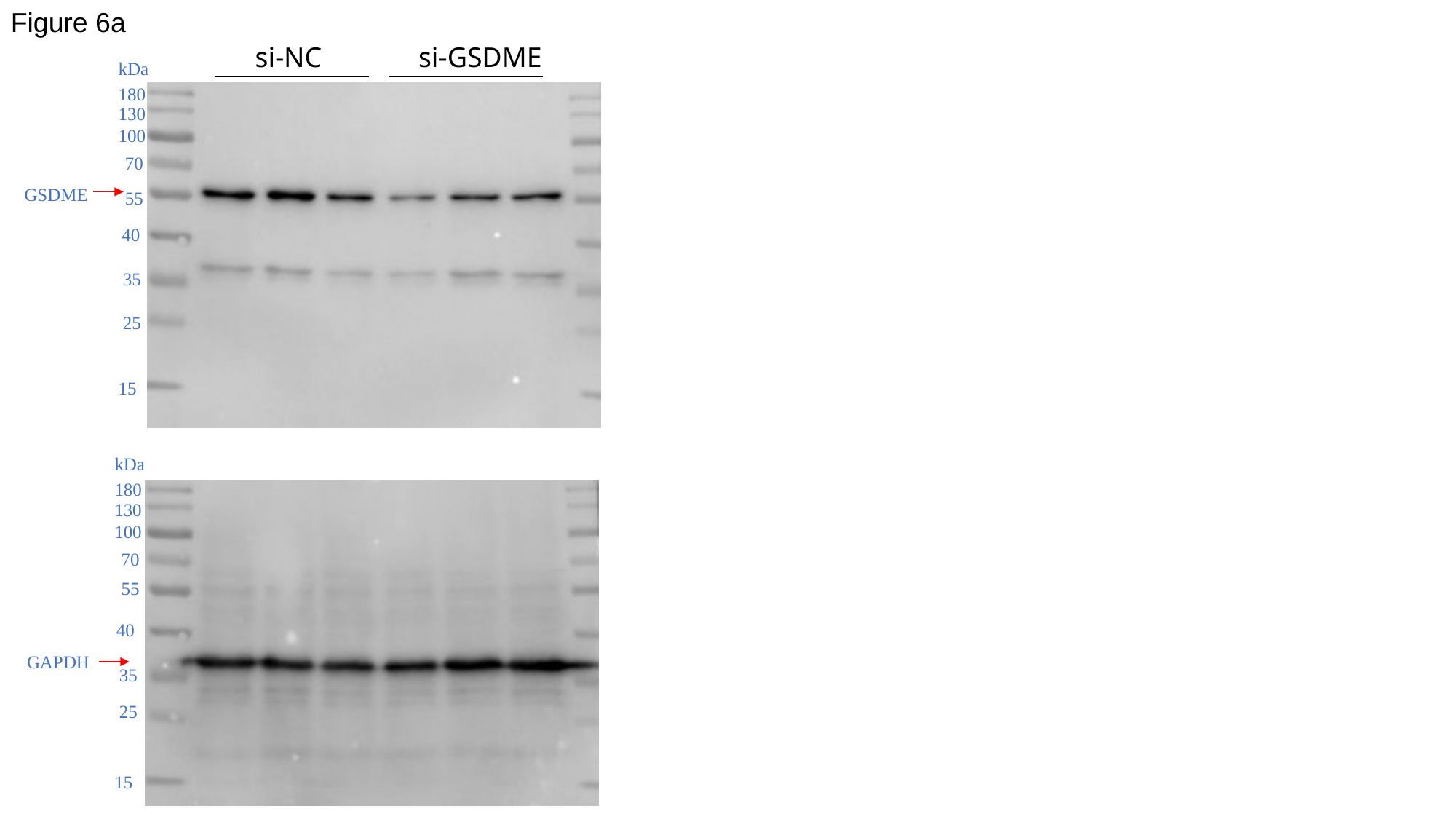

Figure 6a
si-NC
si-GSDME
kDa
180
130
100
70
55
40
35
25
15
GSDME
kDa
180
130
100
70
55
40
35
25
15
GAPDH

## Slide 17
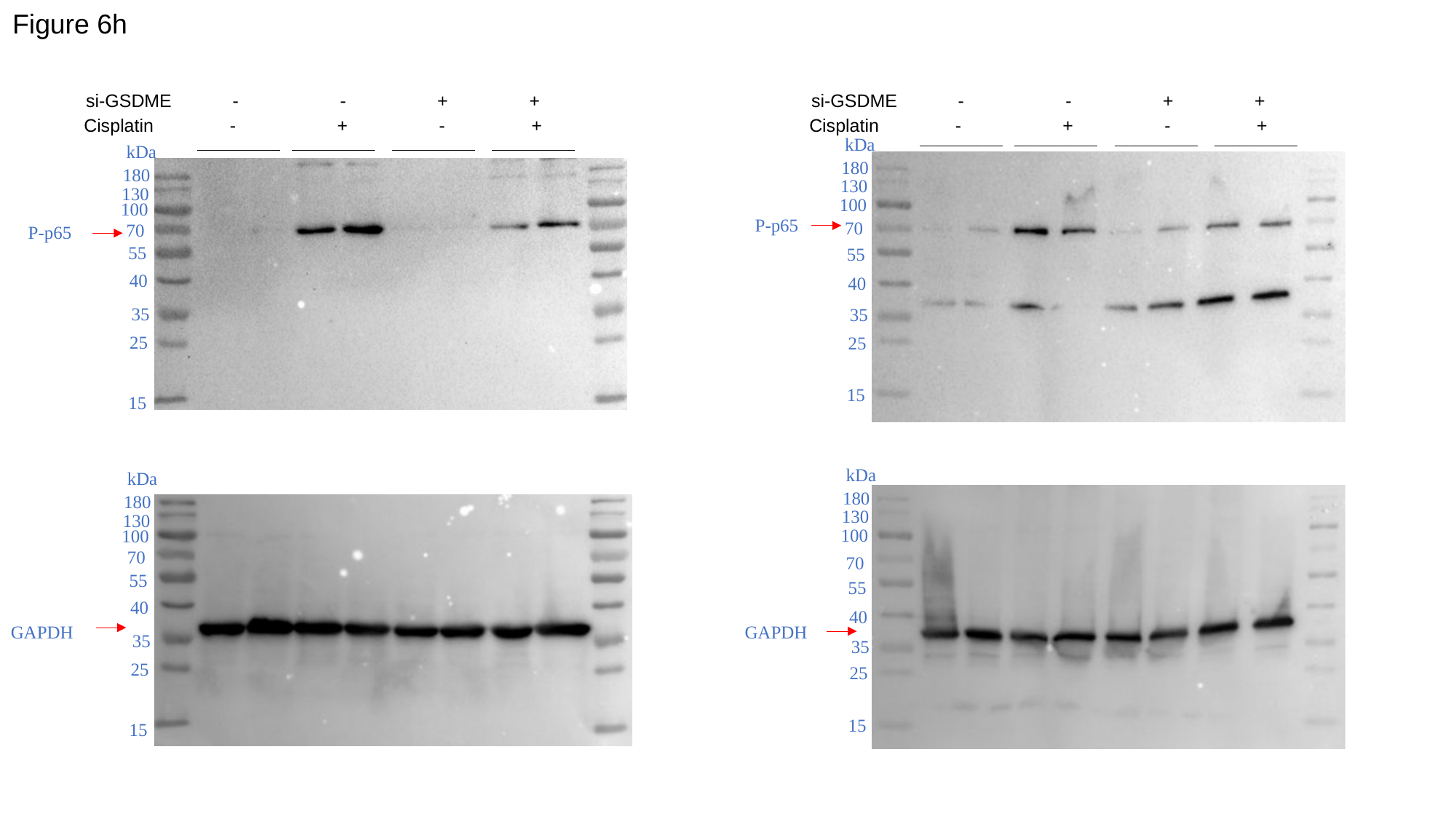

Figure 6h
si-GSDME - - + +
si-GSDME - - + +
Cisplatin - + - +
Cisplatin - + - +
kDa
130
100
70
55
40
35
25
15
180
kDa
130
100
70
55
40
35
25
15
180
P-p65
P-p65
kDa
130
100
70
55
40
35
25
15
180
kDa
130
100
70
55
40
35
25
15
180
GAPDH
GAPDH

## Slide 18
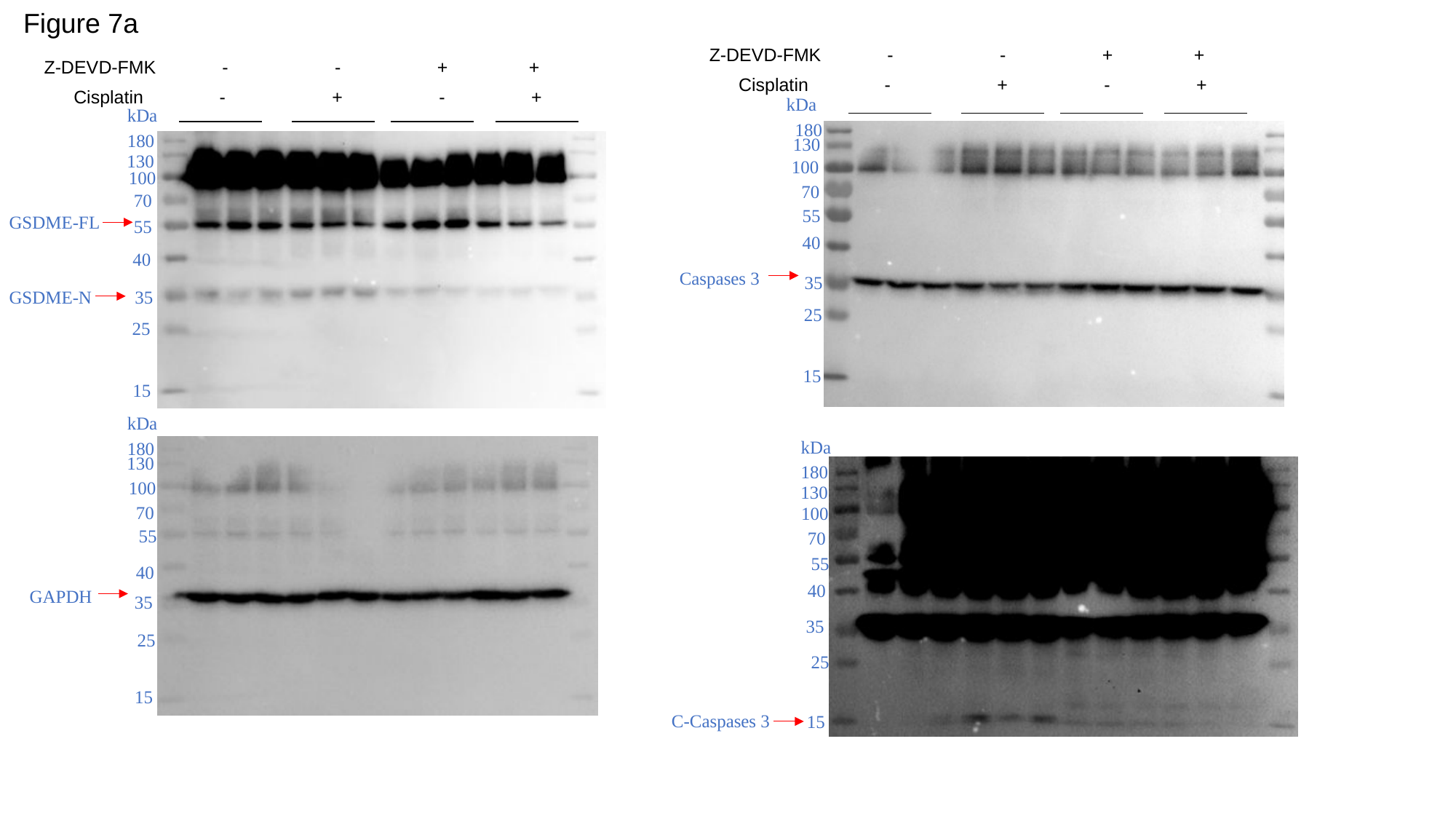

Figure 7a
Z-DEVD-FMK - - + +
Z-DEVD-FMK - - + +
Cisplatin - + - +
Cisplatin - + - +
kDa
130
100
70
55
40
35
25
15
kDa
180
130
100
70
55
40
35
25
15
180
GSDME-FL
Caspases 3
GSDME-N
kDa
180
130
100
70
55
40
35
25
kDa
180
130
100
70
55
40
35
25
GAPDH
15
C-Caspases 3
15

## Slide 19
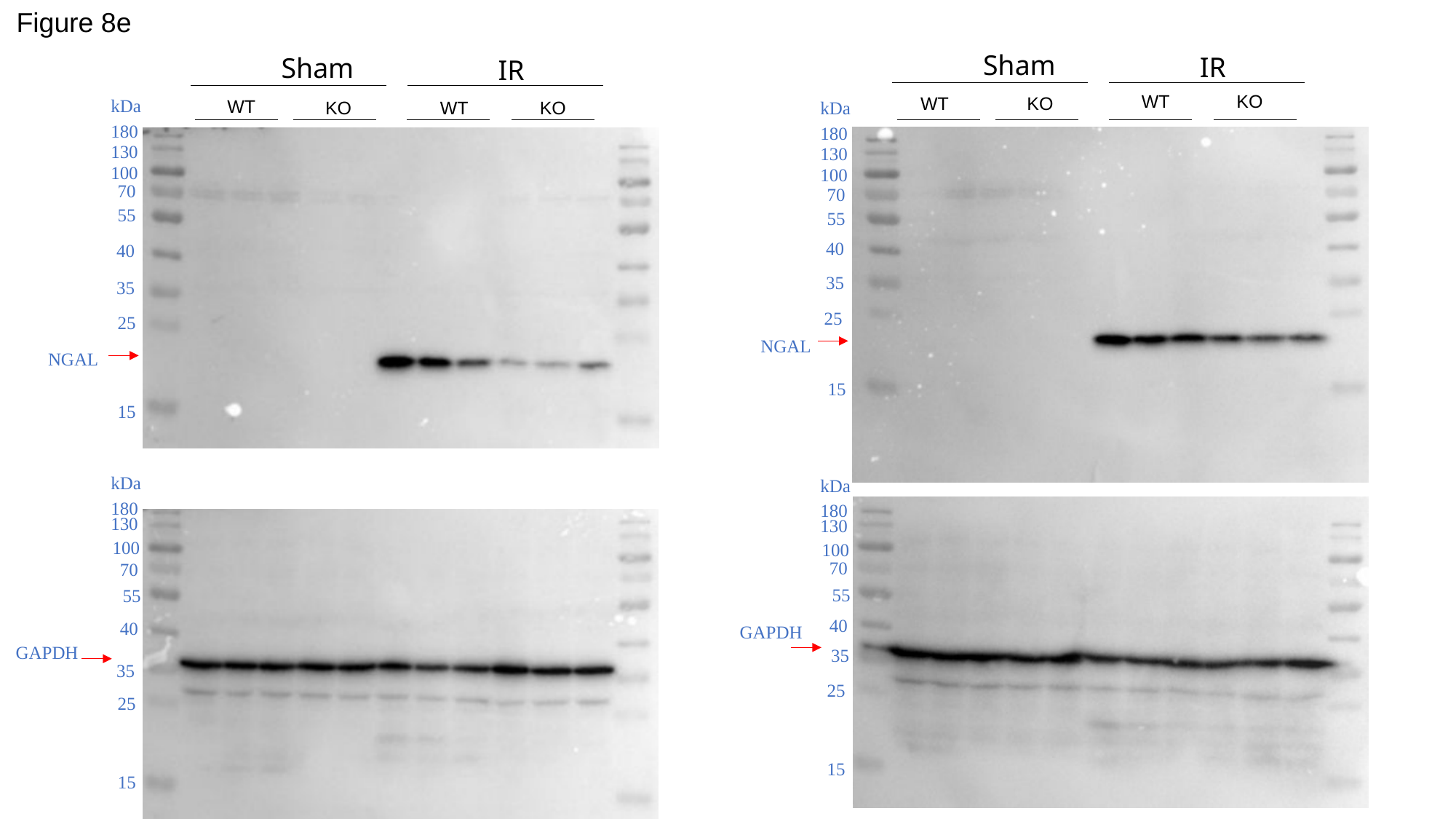

Figure 8e
Sham
IR
Sham
IR
kDa
180
130
100
70
55
40
35
25
15
WT
KO
KO
WT
NGAL
kDa
180
130
100
70
55
40
35
25
GAPDH
15
KO
WT
KO
WT
kDa
180
130
100
70
55
40
35
25
15
NGAL
kDa
180
130
100
70
55
40
35
25
GAPDH
15

## Slide 20
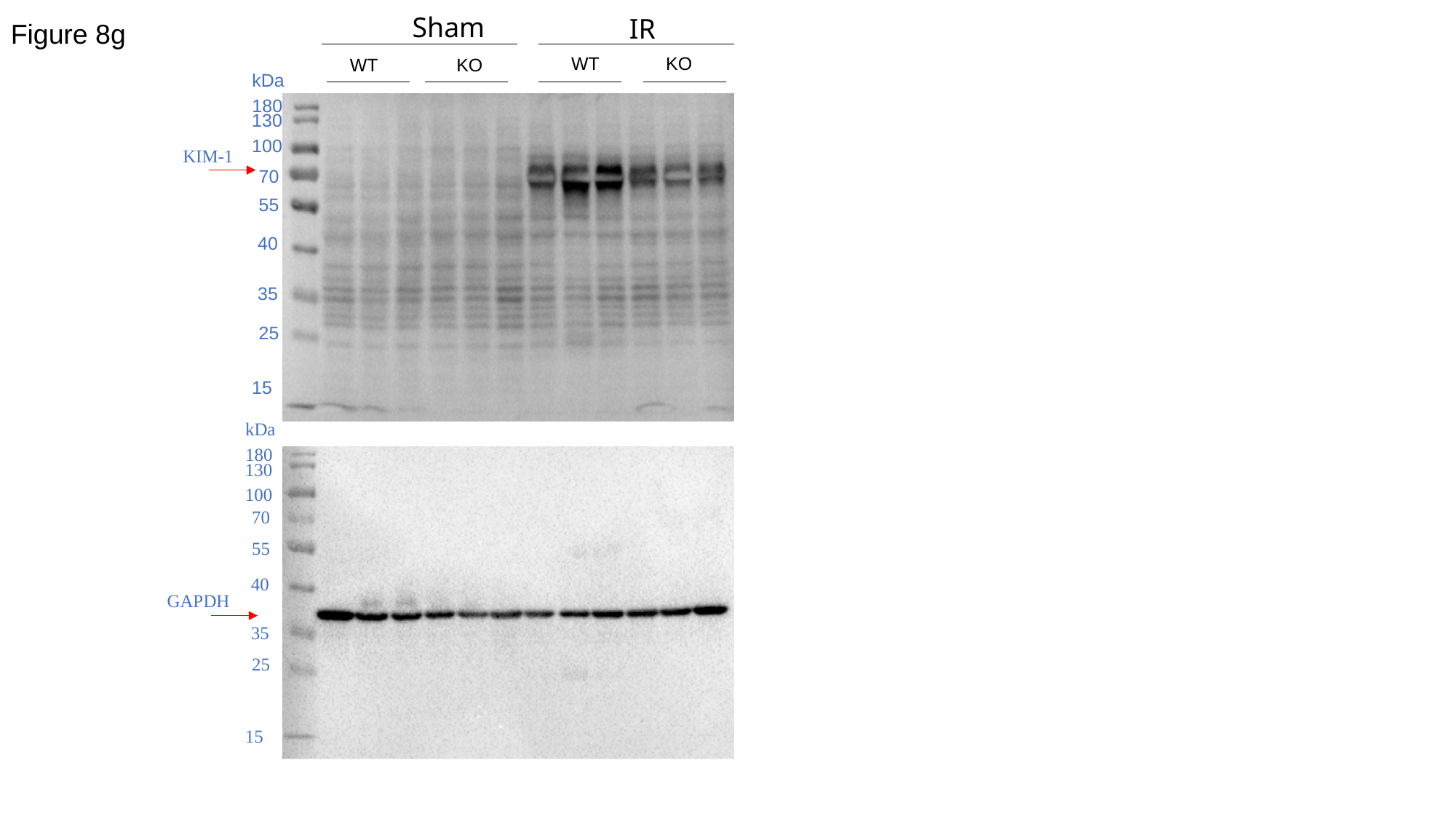

Sham
IR
Figure 8g
KO
WT
KO
WT
kDa
180
130
100
70
55
40
35
25
15
KIM-1
kDa
180
130
100
70
55
40
35
25
15
GAPDH

## Slide 21
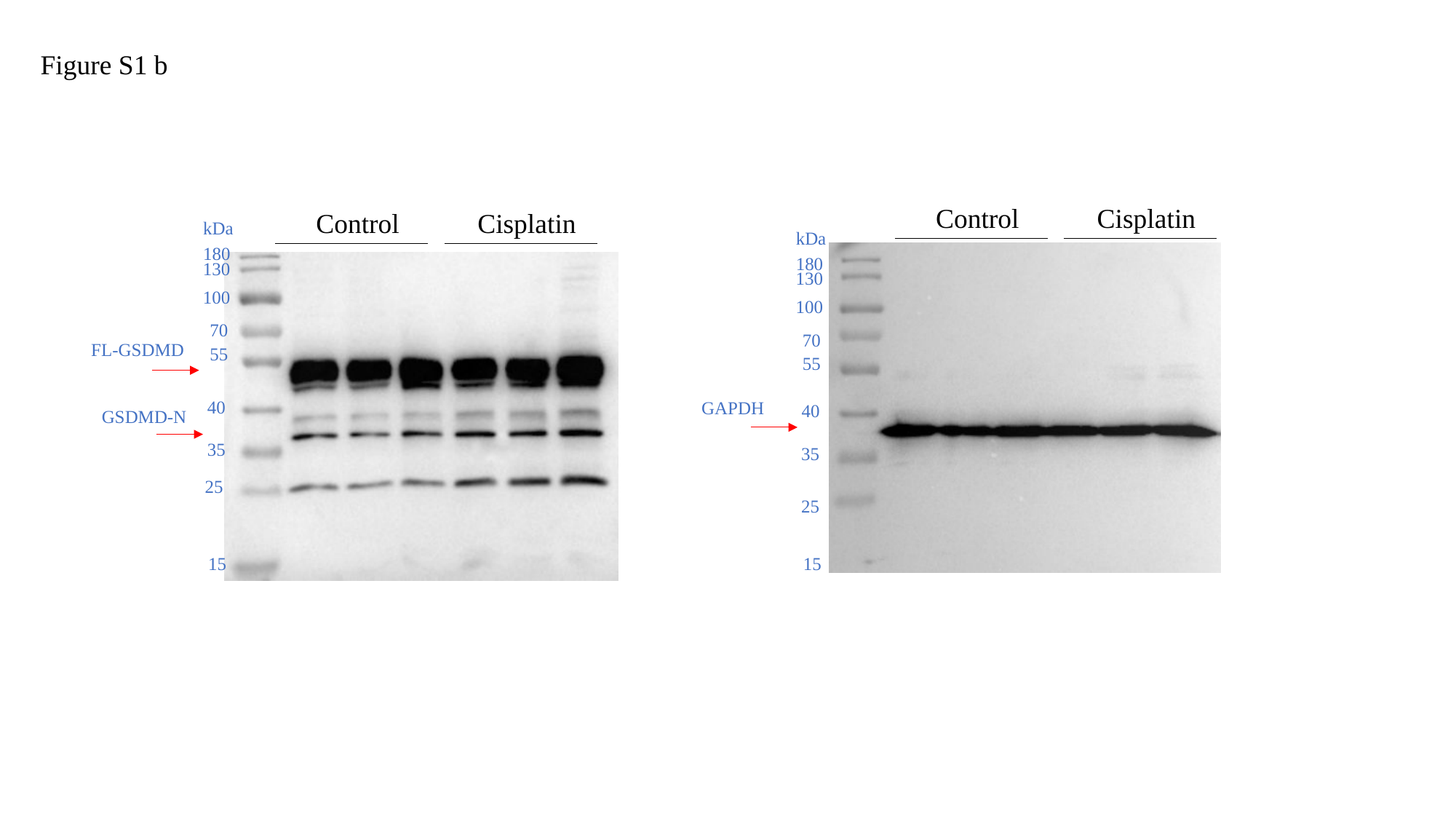

Figure S1 b
Control
Cisplatin
Control
Cisplatin
kDa
180
130
100
70
55
40
35
25
15
kDa
180
130
100
70
55
40
35
25
15
FL-GSDMD
GAPDH
GSDMD-N

## Slide 22
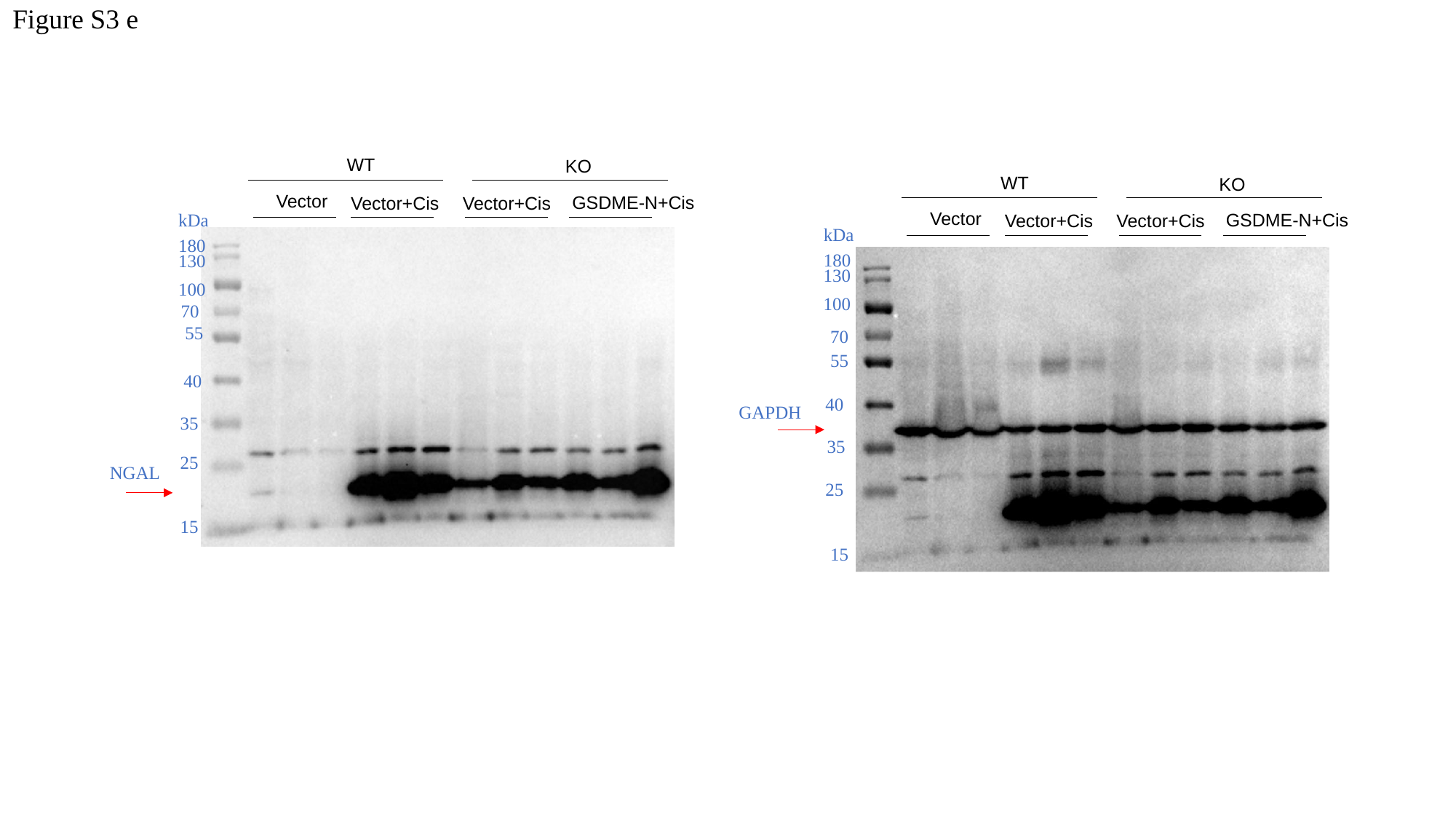

Figure S3 e
WT
KO
WT
KO
Vector
GSDME-N+Cis
Vector+Cis
Vector+Cis
Vector
kDa
180
130
100
70
55
40
35
25
15
GSDME-N+Cis
Vector+Cis
Vector+Cis
kDa
180
130
100
70
55
40
35
25
15
GAPDH
NGAL

## Slide 23
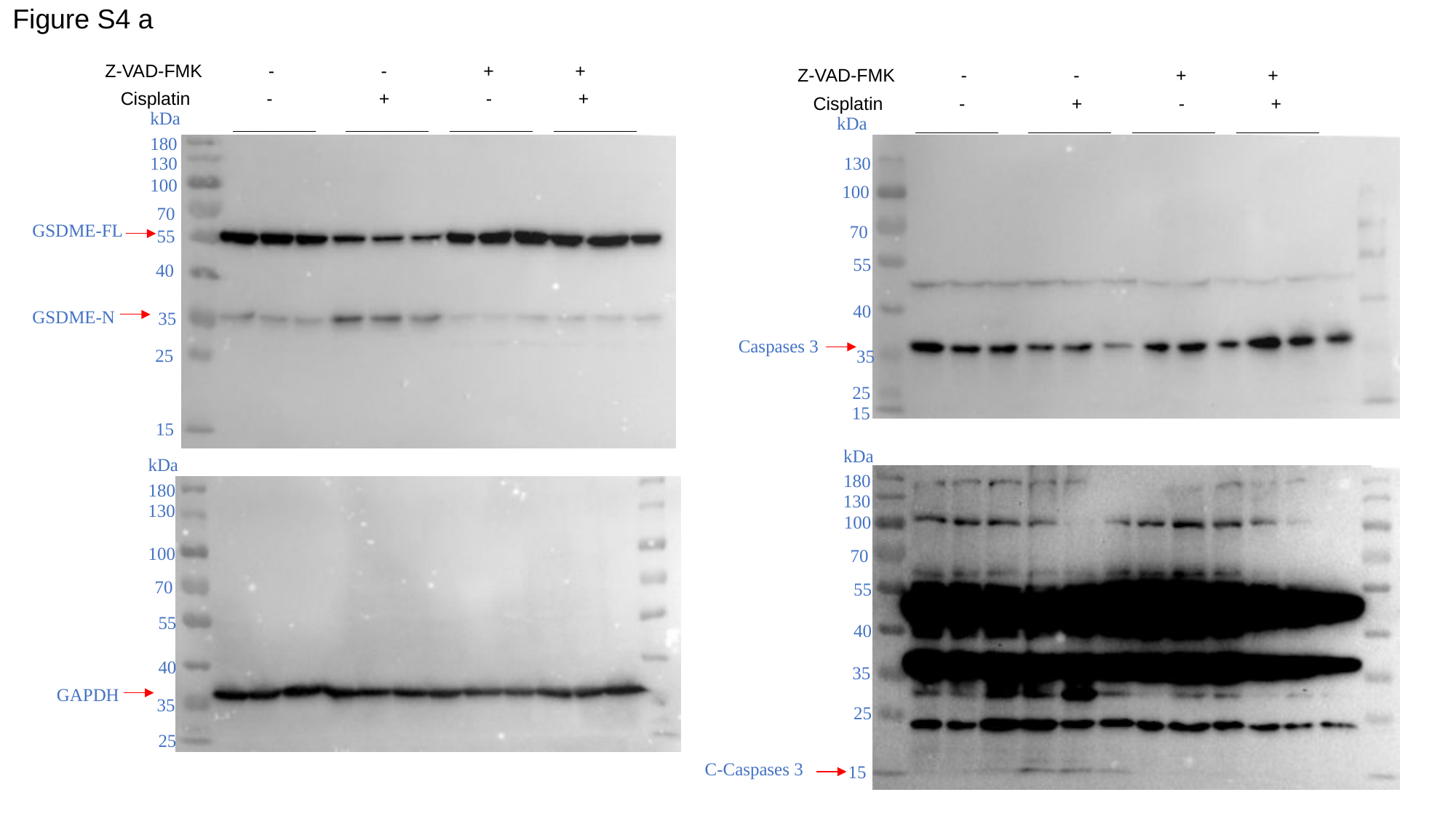

Figure S4 a
Z-VAD-FMK - - + +
Z-VAD-FMK - - + +
Cisplatin - + - +
Cisplatin - + - +
kDa
180
130
100
70
55
40
35
25
15
kDa
130
100
70
55
40
35
25
15
GSDME-FL
GSDME-N
Caspases 3
kDa
180
130
100
70
55
40
35
25
kDa
180
130
100
70
55
40
35
25
GAPDH
C-Caspases 3
15

## Slide 24
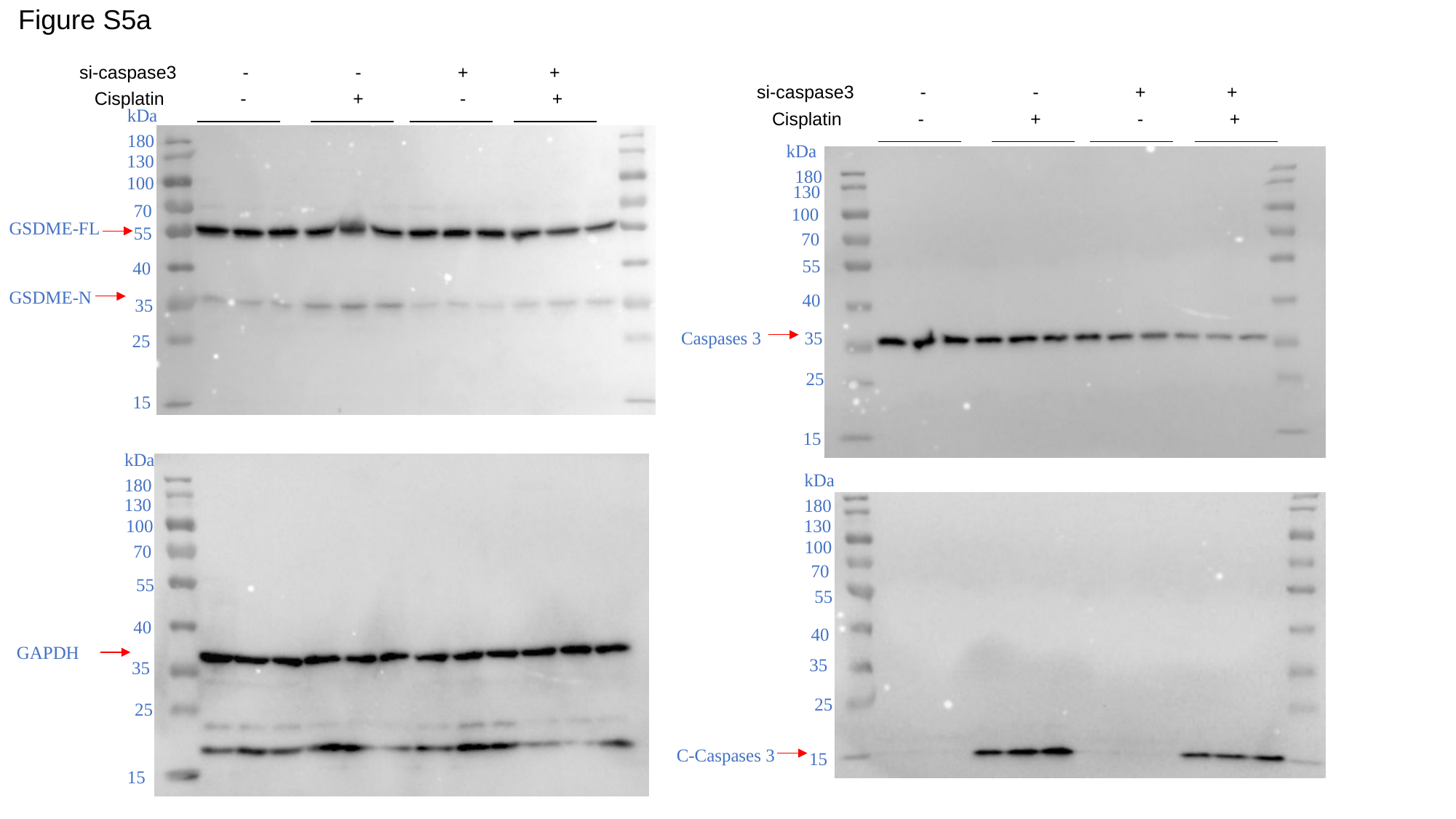

Figure S5a
si-caspase3 - - + +
si-caspase3 - - + +
Cisplatin - + - +
kDa
180
130
100
70
55
40
35
25
15
Cisplatin - + - +
kDa
130
100
70
55
40
35
25
15
180
GSDME-FL
GSDME-N
Caspases 3
kDa
180
130
100
70
55
40
35
25
kDa
180
130
100
70
55
40
35
25
GAPDH
C-Caspases 3
15
15
